# Titrating bacterial growth and chemical biosynthesis for efficient *N*-acetylglucosamine and *N*-acetylneuraminic acid bioproduction

**DOI:** 10.1038/s41467-020-18960-1

**Published:** 2020-10-08

**Authors:** Rongzhen Tian, Yanfeng Liu, Yanting Cao, Zhongjie Zhang, Jianghua Li, Long Liu, Guocheng Du, Jian Chen

**Affiliations:** 1 Key Laboratory of Carbohydrate Chemistry and Biotechnology, Ministry of Education, Jiangnan University, Wuxi, 214122 China; 2 Science Center for Future Foods, Jiangnan University, Wuxi, 214122 China; 3 National Engineering Laboratory for Cereal Fermentation Technology, Jiangnan University, Wuxi, 214122 China

**Keywords:** Metabolic engineering, Metabolic engineering, Applied microbiology

## Abstract

Metabolic engineering facilitates chemical biosynthesis by rewiring cellular resources to produce target compounds. However, an imbalance between cell growth and bioproduction often reduces production efficiency. Genetic code expansion (GCE)-based orthogonal translation systems incorporating non-canonical amino acids (ncAAs) into proteins by reassigning non-canonical codons to ncAAs qualify for balancing cellular metabolism. Here, GCE-based cell growth and biosynthesis balance engineering (GCE-CGBBE) is developed, which is based on titrating expression of cell growth and metabolic flux determinant genes by constructing ncAA-dependent expression patterns. We demonstrate GCE-CGBBE in genome-recoded *Escherichia coli* Δ321AM by precisely balancing glycolysis and *N*-acetylglucosamine production, resulting in a 4.54-fold increase in titer. GCE-CGBBE is further expanded to non-genome-recoded *Bacillus subtilis* to balance growth and *N*-acetylneuraminic acid bioproduction by titrating essential gene expression, yielding a 2.34-fold increase in titer. Moreover, the development of ncAA-dependent essential gene expression regulation shows efficient biocontainment of engineered *B. subtilis* to avoid unintended proliferation in nature.

## Abstract (web summary)

An imbalance between cell growth and bioproduction of engineered microbes often reduces production efficiency. Here, the authors report genetic code expansion-based cell growth and biosynthesis balance engineering to achieve high levels production of N-acetylglucosamine in *E. coli* and N-acetylneuraminic acid in *B. subtilis*.

## Introduction

**Fig. 1:**
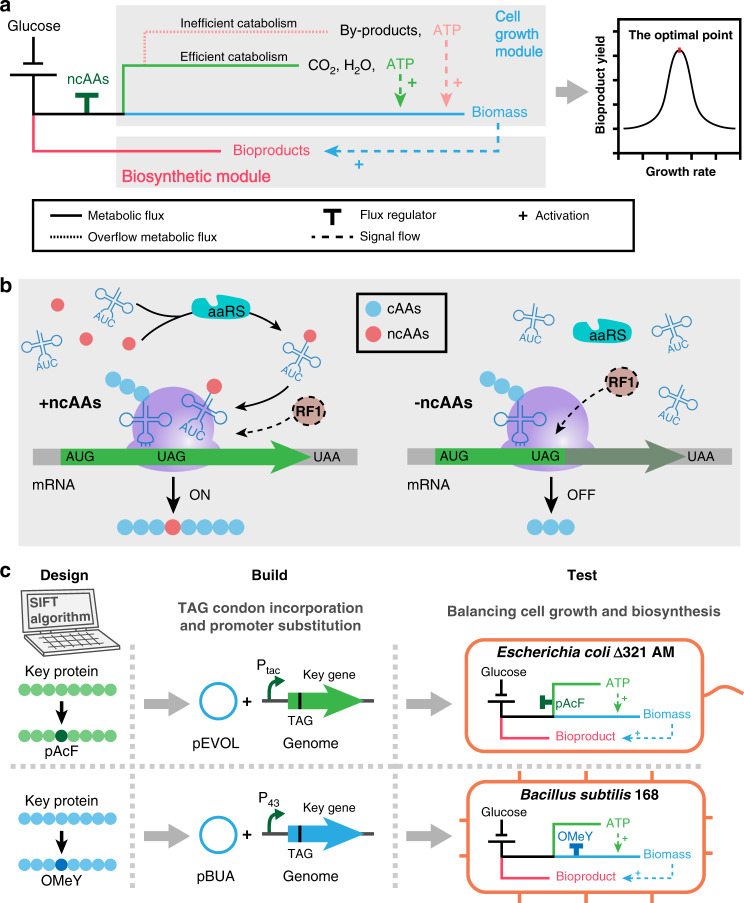
Balancing cell growth and product synthesis based on genetic code expansion. **a** Metabolic flux distribution during cell growth and biosynthesis. Theoretically, the balance between cell growth and product synthesis can be achieved by regulating the distribution of metabolic fluxes to obtain optimal yield. **b** Utilization of the non-canonical amino acid (ncAA) incorporation tool to build a molecular switch. Translation can be interrupted when an amber stop codon (TAG) is inserted into the gene sequence of interest (green arrow) in the absence of ncAAs. However, after ncAAs are added, aminoacyl-tRNA synthetase (aaRS) can catalyze ncAA-loading of tRNAs (blue clover-leaf structure), which are able to restore gene expression. While peptide chain release factor RF1 was depleted in genetically recoded *E. coli* ∆321AM, competition between RF1 and aminoacyl-tRNA molecules may have reduced the efficiency of the ncAA system in non-genome-recoded *B. subtilis* 168 (the dotted circle). **c** Workflow of the GCE-CGBBE strategy in *E. coli* and *B. subtilis*. First, amber stop codon (TAG) substitution sites of key genes that control cell growth were designed based on the SIFT algorithm. Next, strains harboring ncAA-incorporation tools were constructed to achieve regulatory expression of genes with amber stop codon insertion. Finally, cell growth and product balance were tested in two of the best-studied Gram-negative and Gram-positive bacteria, i.e., genetically recoded *E. coli* Δ321AM and non-genome-recoded *B. subtilis* 168. cAAs, canonical amino acids.

Metabolic engineering is an enabling technology for developing sustainable biomanufacturing processes for chemicals, fuels, and nutraceuticals using renewable feedstock by repurposing cellular metabolism^1–4^. High-level bioproduction of 1,3-propanediol and 1,4-butanediol demonstrates the success of bio-based chemical production^5–7^. However, metabolic fluxes in target chemical biosynthetic pathways need to be shifted from cellular endogenous metabolism for cell growth to bioproduction, which conflicts with a cell’s need for ensuring survival^3^. When redirection of target metabolic pathways is insufficient, abundant substrate is mainly used for cell growth, which is often accompanied by by-product synthesis, resulting in low production yield and titer. In contrast, excessive shunting affects cell growth, and subsequently reduces productivity (Fig. 1a)^8^. Therefore, balancing cell growth with biosynthesis is one of the core issues in constructing highly efficient metabolically engineered strains^9,10^.

Efforts have been made to solve these issues through various metabolic engineering strategies, such as de-coupling cell growth with biosynthesis based on genetic circuits, co-coupling cell growth with biosynthesis by coupling production to overall energy and biomass formation, and parallel metabolic pathway engineering via separating bioproduction from cell growth by implementing parallel dual carbon sources pathways^11–13^. De-coupling cell growth with biosynthesis commonly uses toggles, autonomous and quorum-sensing system-based genetic circuits, or optogenetic circuits to shift growth and production phases^11,14–17^. However, a potential interference of intracellular metabolites or the culture environment with genetic circuits and the requirement of large-scale fermentation equipment redesign for optogenetic circuit implementation impact either orthogonality or large-scale applicability^18,19^. Moreover, incorporating synthetic regulatory circuits into native pathways requires extensive genome editing, the designing and building of which is challenging^20^. Co-coupling cell growth with biosynthesis is suitable for obtaining sufficient titer, but difficult to use for improving yield, due to difficulties in regulating cell growth, especially for biosensor-based cell growth regulation that often shows atypical physiological properties^21,22^. Although parallel metabolic pathway engineering improves production yields, the lack of precise cell growth regulation causes overflowing metabolism, resulting in by-product (acetate) formation^13^. Moreover, parallel metabolic pathway engineering could be limited by precursor availability depending on multiple endogenous pathways, owing to an increased complexity of pathway designs, and the metabolic burden of introducing multiple substrate utilization pathways^23,24^. Designing a generic and robust cell growth and biosynthesis balancing strategy is challenging.

Metabolic connections between cell growth and target product biosynthesis increase the complexity of balancing cellular metabolic pathways. Therefore, an orthogonal gene expression system should regulate target metabolic pathways to avoid interference by other pathways. For example, the genetic code expansion (GCE)-based orthogonal protein translation system introduces orthogonal aminoacyl-tRNA synthetase (aaRS)/tRNA pairs into a host to incorporate non-canonical amino acids (ncAAs) rather than the 20 proteinogenic amino acids into proteins by reassigning non-canonical codons to ncAAs^25^. More than 200 ncAAs can be site-specifically incorporated into proteins, thereby expanding the genetic code of microorganisms, which lays the foundation for expanding functional diversity of proteins and the construction of orthogonal protein translation systems (Fig. 1b)^25–29^. The amber stop codon (TAG) is least frequently used in bacteria, and it has only one corresponding release factor; therefore, it is usually used in orthogonal tRNA design to minimize direct impacts on the expression levels of non-target genes. Recent progress in genome engineering and writing has enabled the construction of genetically recoded *Escherichia coli*, including *E. coli* with all amber stop codons replaced by ochre stop codons (TAA), or with a 61-codon genome (serine codons (TCG and TCA) and amber stop codons were replaced by their synonyms AGC, AGT, and TAA, respectively), which provides a favorable chassis cell for applying GCE-based orthogonal translation systems by reassigning free codons to ncAAs^30,31^. The expression of genes with amber stop codon insertions requires ncAAs, and their expression levels are presumed ncAA-dependent. At the same time, tunable translation control tools using site-specific ncAA incorporation have been studied^29,32–34^. Therefore, by inserting amber stop codons into genes that are important for controlling cell growth, such as genes involved in central carbon metabolic pathways, should be regulated by ncAA abundances. Further titration of ncAAs to determine optimum concentrations to precisely control cell growth rates can potentially be used for balancing cell growth and bioproduction (Fig. 1a, c).

In this study, a GCE-based cell growth and biosynthesis balancing engineering (GCE-CGBBE) strategy for efficient bioproduction is developed, which is based on titrating expression of cell growth and metabolic flux determinant genes by constructing ncAA-dependent expression patterns (Fig. 1c). A translation system for incorporating the ncAAs into proteins via amber stop codon insertion is optimized for genetically recoded *E. coli* and non-genome-recoded *Bacillus subtilis*, two of the best-studied Gram-negative and Gram-positive bacteria, respectively. We demonstrate GCE-CGBBE in genome-recoded *E. coli* by precisely balancing glycolysis and *N*-acetylglucosamine (GlcNAc) production. We further expand GCE-CGBBE to non-genome-recoded *B. subtilis* to improving *N*-acetylneuraminic acid (NeuAc) production. In addition, the development of ncAA-dependent essential gene expression regulation shows efficient biocontainment of engineered *B. subtilis*.

## Results

### Testing the ncAA-incorporation system in *E. coli* Δ321AM

The orthogonal translation system for ncAA incorporation is composed of mutant aaRS/tRNA pairs for translating non-canonical codons, which has been well established in *E. coli*^35^. Therefore, the widely used pEVOL-based ncAA/ *p*-acetyl-L-phenylalanine (pAcF) incorporation system was selected, which is specific for translating amber stop codons and have only limited activity for other 20 proteinogenic amino acids^35^. In our pAcF incorporation system, we selected the *E. coli* ∆321AM strain, which has been genetically recoded by (1) exchanging all 321 amber stop codons in the genome with ochre stop codons for translation termination; (2) knockout of peptide chain release factor RF1 to avoid competition between RF1 and the orthogonal tRNA for efficient translation; (3) recovering almost the same cell growth rate as wild type *E. coli* via adaptive evolution^30,36^. Therefore, *E. coli* Δ321AM was suitable for specifically using the amber stop codon for incorporating ncAAs.

**Fig. 2:**
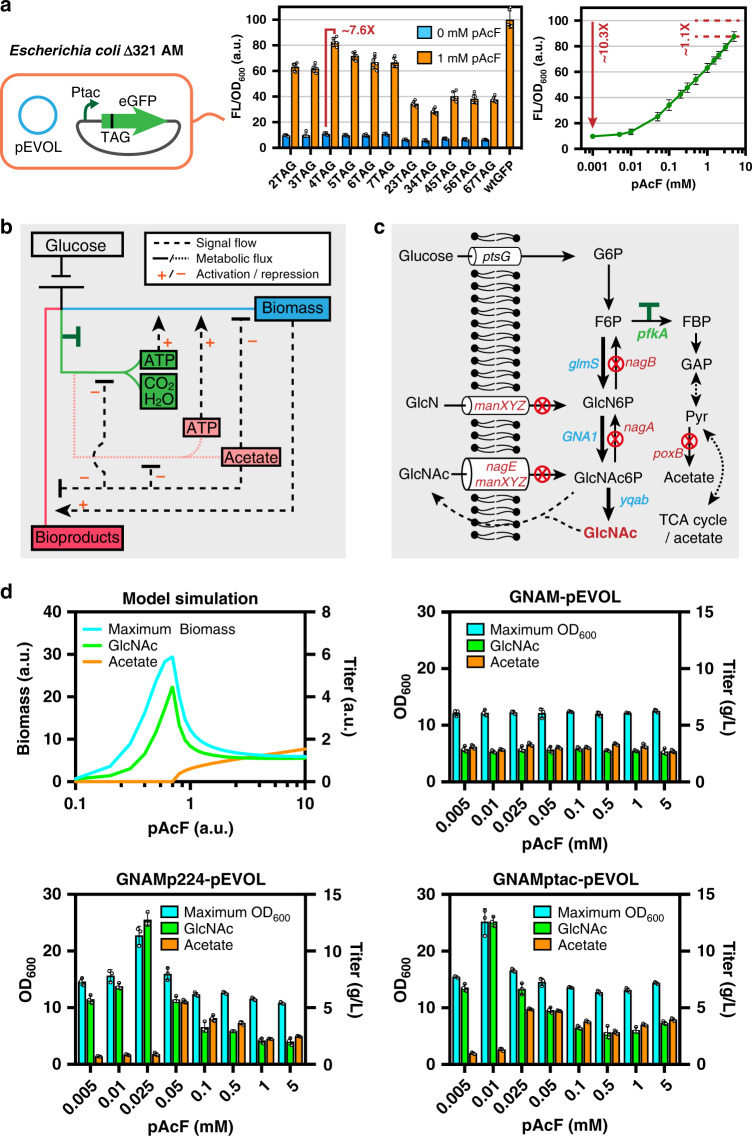
Non-canonical amino acid (ncAA) incorporation tool-based pathway regulation in genetically recoded *Escherichia coli* Δ321AM. **a** Testing the efficiency of ncAA tool-pEVOL using the reporter protein GFP (left panel). The histogram (middle panel) represents the degree of GFP expression recovery after replacing one or two original codons by amber stop codons in the sequence from the second to the seventh codon (2TAG to 7TAG and 23TAG to 67TAG). The data are expressed as the mean ± SD from six (*n* = 6) biologically independent replicates. The line graph (right panel) shows the use of the 2TAG GFP gene to test GFP expression levels in the presence of different concentrations of *p*-acetyl-L-phenylalanine (pAcF). The data are expressed as the mean ± SD from three (*n* = 3) biologically independent replicates. **b** Intracellular metabolic feedback loop of engineered *E. coli*. ‘T’ marker represents the regulation of metabolic flow. **c** Construction of the *N*-acetylglucosamine (GlcNAc) biosynthetic *E. coli* Δ321AM strain. Red circular symbols represent gene knockouts, the ‘T’ marker represents the regulation of metabolic flux, and bold arrows represent an increase in metabolic flux. **d** Model simulated (half-saturation constant *K*_S_ = 15) and actual fermentation results of the three GlcNAc biosynthetic strains measured in the presence of different pAcF concentrations. The data are expressed as the mean ± SD from three (*n* = 3) biologically independent replicates. Source data underlying (**a**), (**d**) are provided as a Source data file.

Next, the effects of replacing amber stop codons at different positions in the N-terminal coding region on the dynamic range of gene expression in the presence or absence of pAcF were tested using green fluorescent protein (GFP) under the control of the P_*tac*_ promoter. By replacing one or two original codons from second to seventh position with amber stop codons (six mutants with a single amber stop codon replacement (2TAG to 7TAG), and five mutants with double adjacent amber stop codons replacement (23TAG to 67TAG)), it was found that the pEVOL-pAcF system can enhance gene expression by 6.48-fold and 7.65-fold in *E. coli* Δ321AM when the second (2TAG) and fourth (4TAG) codon of the GFP coding sequence are replaced, respectively. Furthermore, we selected the gene of 2TAG GFP to test the regulation range of gene expression by adding different concentrations of pAcF (0.001–5 mM). With increasing pAcF concentration, the fluorescence intensity increased logarithmically, achieving a 9.01-fold enhancement of gene expression (Fig. 2a). In addition, the cytotoxicity of pAcF for *E. coli* Δ321AM harboring pEVOL was also tested, showing that the addition of different concentrations of pAcF had no significant effect on cell growth (Supplementary Fig. 1). Tests on the ncAA-incorporation tool indicated that the gene expression level was dependent on pAcF concentration, which laid the foundation for its further application in titrating gene expression in *E. coli* Δ321AM.

### Balancing *E. coli* growth and GlcNAc biosynthesis

In bioproduction systems, cells are essential biocatalysts for improving production efficiency. However, excess or improper control of cell growth often causes decreased production yield or by-product accumulation. When *E. coli* is provided with glucose in excess, high growth rates cause an overflow of the glycolytic pathway, accompanied by acetate synthesis and ATP production^37^. Although ATP produced in the acetate synthesis pathway may be beneficial for intracellular thermodynamically unfavorable reactions, thereby improving cell growth, acetate accumulation seriously impairs cell growth and substrate to target product conversion (Fig. 2b). Therefore, balancing cell growth and target compound biosynthesis is a win-win strategy, which cannot only balance the competition between cell growth and target compound synthesis, but also serves to avoid the formation of overflow by-products.

Consequently, biosynthesis of GlcNAc, a widely used nutraceutical in cartilage protection and arthritis treatment, was chosen as an example, which competes for both carbon flux in the glycolytic pathway and precursors of cell wall synthesis (Fig. 2c). Using *E. coli* Δ321AM as the initial host, we first constructed the GlcNAc-producing strain GNAM by knocking out seven genes (GlcNAc degradation-related genes *nagABE* and *manXYZ*, and pyruvate oxidase *poxB*), and by strengthening the GlcNAc biosynthetic pathway through integrated expression and promoter optimization of three pathway genes, i.e., *glmS*, *GNA1*, and *yqaB*, which originated from *E. coli* K-12 *glmS* mutant 2123-72 with a moderately strong promoter (P_566_), *Saccharomyces cerevisiae* S288C *GNA1* with a strong promoter (P_*tac*_), and *E. coli* K-12 *yqaB* with a strong promoter (P_*tac*_), respectively (Supplementary Figs. 2, 3)^38,39^. The GNAM strain produced 2.81 g/L of GlcNAc and 3.13 g/L of acetate (Supplementary Fig. 3). To aid the design of a balancing strategy, we constructed a kinetic model of GlcNAc production and cell growth of the engineered strains. In this model, the pAcF concentration represented a controller of the glycolytic flux, which controlled the expression of phosphofructokinase by inserting an amber stop codon into the corresponding *pfkA* gene. When ncAA was added in excess in silico, excess glycolytic flux enabled rapid cell growth, and activated the synthesis of acetate and ATP; consequently, with an accumulation of acetate, bacterial growth was rapidly inhibited. As pAcF was gradually decreased in silico, the glycolytic flux decreased, which slowed acetate accumulation, and allowed for increased cell division. Therefore, more GlcNAc was produced with increasing numbers of cells as biocatalysts. As the concentration of pAcF continued to decrease in silico, the carbon flux of the glycolytic pathway gradually decreased to limited cell growth and further reduced production (Fig. 2d; Supplementary Fig. 4). Through model simulations, we found that there should be a balance between cell growth and product synthesis, and GlcNAc can be efficiently biosynthesized within a narrow pAcF concentration range around the balance point.

In order to verify the above hypothesis, the *pfkA* gene of *E. coli* was selected for modulation, because its expression determines the carbon flux of both glycolytic and GlcNAc biosynthetic pathways. First, pEVOL was transformed into GNAM to obtain GNAM-pEVOL. Next, an amber stop codon was inserted after the translation start codon (ATG) of the *pfkA* coding sequence in the genome of the engineered strain. At the same time, in order to increase transcription and subsequently reduce the working concentration of pAcF, we replaced the promoter of *pfkA* with strong P_224_ and P_*tac*_ promoters, yielding GNAMp_224_-pEVOL and GNAMp_*tac*_-pEVOL strains, respectively. Therefore, *pfkA* expression could be regulated by titrating pAcF in the medium, further fine-tuning the carbon flux between glycolytic and GlcNAc biosynthetic pathways.

Indeed, different concentrations of pAcF influenced both cell growth and GlcNAc biosynthesis. Compared with engineered *E. coli* without balanced growth and bioproduction, both cell growth and GlcNAc biosynthesis were greatly improved with decreased acetate. The GNAMp_*tac*_-pEVOL strain grown with 0.01 mM pAcF resulted in a 2.06-fold increase in maximal OD_600_ (25.21), 83.75% decreased acetate levels (5.54 vs. 0.90 g/L), and a 4.54-fold increased GlcNAc titer (12.77 g/L) with 0.48 g_GlcNAc_/g_glucose_, reaching 88.52% of the theoretical pathway yield (Fig. 2d, Supplementary Fig. 5). In order to ensure that the strategy is not only effective at high concentrations of glucose, fermentation experiments were also performed at a gradient glucose concentration ranging from 5 to 30 g/L. Strain GNAMptac-pEVOL showed higher GlcNAc titer and lower acetate titer regardless of the glucose concentration, indicating the effectiveness of the strategy under different culture conditions (Supplementary Fig. 6). Besides, the expression level of PfkA was also tested by monitoring fluorescence of GFP fused with PfkA, which increased logarithmically with an increase of pAcF concentration as expected (Supplementary Fig. 7).

### *B. subtilis* expression system for incorporating *O*-methyl-L-tyrosine

**Fig. 3:**
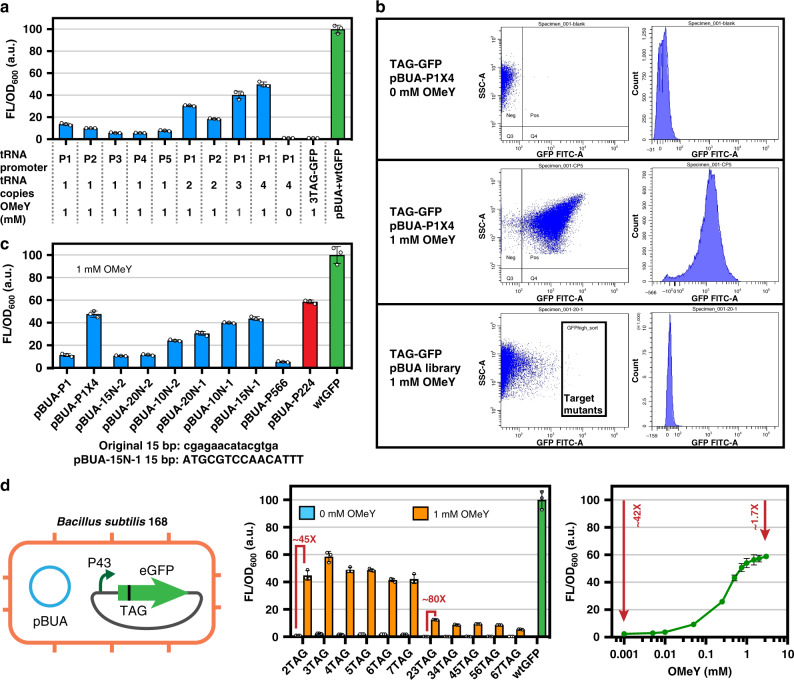
Construction and optimization of *Bacillus subtilis* non-canonical amino acid (ncAA) incorporation tools. **a** Optimization of tRNA promoter and copy number. **b** Screening of three random mutant libraries for efficient pBUA mutants using flow cytometry. SSC-A: granularity, FITC-A: GFP fluorescence. **c** Degree of GFP expression level recovery by efficient pBUA mutants obtained from three random mutant libraries, harboring pBUA constructs with synthetic promoters. **d** Efficiency verification of pBUA in *B. subtilis*. The histogram (left panel) represents the degree of GFP expression recovery after replacing one or two original codons with amber stop codons in the sequence from the second to the seventh codon (2TAG to 7TAG and 23TAG to 67TAG). The line graph (right panel) shows the use of the 3TAG GFP gene to test GFP expression levels in the presence of different concentrations of *p*-acetyl-L-phenylalanine (pAcF). All the data are expressed as the mean ± SD from three (*n* = 3) biologically independent replicates. Source data underlying (**a**), (**c**), (**d**) are provided as a Source data file.

In order to further verify the universality of the GCE-CGBBE strategy for non-genetically recoded bacteria, the strategy was further expanded to the non-genome-recoded Gram-positive model bacterium *B. subtilis* to balance growth and bioproduction. Since ncAA-incorporation tools are lacking for *B. subtilis*, an ncAA *O*-methyl-L-tyrosine (OMeY) incorporation tool for *B. subtilis*, pBUA, was first constructed and tested. We selected an aaRS/tRNA mutant pair derived from *Methanococcus jannaschii* that can incorporate OMeY into amber stop codons^25^. The coding gene of the mutant aaRS/tRNA pair for OMeY incorporation was inserted into pHT01 plasmid for expression. Accordingly, successful expression of genes with amber stop codon insertion should be observed in the presence of OMeY. Therefore, efficiency of the OMeY incorporation tool (pBUA) was tested by comparing fluorescence intensities of engineered *B. subtilis* harboring a GFP gene with or without amber stop codon substitution. The higher the percentage of GFP gene expression with TAG substitution compared to that without TAG substitution, the more efficient the OMeY incorporation tool. The first version of pBUA tools (pBUA-P1 to pBUA-P5) was constructed based on the pHT01 plasmid by integrating aaRS and mutant tRNA gene copies under the control of P_43_ or one of five original *B. subtilis* tRNA promoters (P1 to P5), respectively (Fig. 3a). When one copy of the tRNA mutant was under the control of a P1 or P2 promoter, the expression level of GFP with TAG substitution (at the third codon in the N-terminal region) reached 13.63%, and only 10.04% of GFP expression without TAG substitution, which showed that P1 and P2 were more efficient than the other original *B. subtilis* tRNA promoters. To further enhance OMeY incorporation efficiency, copy number and promoter selection were optimized for this tRNA mutant. As four copies of the tRNA mutant were under the control of the P1 promoter, an effective OMeY incorporation tool, pBUA-P1X4, was constructed to restore ~49.78% of GFP expression with only 2.21% leakage (Fig. 3a). For promoter optimization of *tRNA* genes, two strategies were used. The first strategy was to construct random mutation libraries to screen promoters for efficiently driving tRNA expression. We constructed three random mutation libraries (pBUA-10N, pBUA-20N, and pBUA-15N) based on pBUA-P1 by random A, T, G, and C insertions 10 bp upstream of the tRNA coding sequence, 20 bp upstream of the tRNA coding sequence, and 15 bp between the −35 and −10 regions of promoter P1 via degenerate primer design and PCR. After screening 6,000,000 cells using flow cytometry, and further characterization of 1000 strains obtained from each library, the most efficient pBUA mutant in all libraries was finally obtained (Fig. 3b). The efficiency of the pBUA system with a single tRNA mutant copy obtained by this strategy was 2.87-fold higher than that of pBUA-P1, which restored the expression level of GFP to 43.65%. The second strategy was to rationally replace promoter P1 with strong synthetic promoters to enhance tRNA mutant expression. The strong promoters P_224_ and P_566_ of *B. subtilis* were selected to express mutant tRNAs using the pBUA system. The pBUA-P_224_ construct further restored GFP levels to 58.50%, which was 1.23-fold higher than that of the four tRNA copies with the P1 promoter system that was selected for OMeY incorporation in *B. subtilis* (Fig. 3c).

We then tested the effects of different amber stop codon insertion sites on the dynamic range of expression of pBUA-P_224_ in the presence or absence of OMeY. By replacing one or two codons from the second to seventh position with amber stop codons (six mutants with a single amber stop codon replacement (2TAG to 7TAG), and five mutants with double adjacent amber stop codon replacements (23TAG to 67TAG)), it was found that pEBUA-P_224_ can activate up to 77.75-fold gene expression when the second and third codons of the GFP coding sequence were replaced (23TAG), while GFP expression was difficult to detect in the absence of OMeY, demonstrating that the corresponding tRNA was charged exclusively with the reassigned amino acid by an aaRS that did not interact with any other tRNA. In parallel, when the third codon was replaced with TAG, the expression level of GFP could be maximally rescued, reaching 58.50% of GFP without TAG codon insertion. Furthermore, we selected the gene of 3TAG GFP and tested the regulation range of gene expression by adding different concentrations of OMeY (0.001–3 mM). Fluorescence intensity increased with increasing OMeY concentration, achieving 24.82-fold activation of gene expression (Fig. 3d; Supplementary Fig. 8). In addition, the cytotoxicity of the OMeY incorporation system was tested by culturing *B. subtilis* harboring pBUA-P_224_ at different concentrations of OMeY. This showed that the OMeY incorporation system had no significant effect on cell growth in the OMeY concentration range tested (Supplementary Fig. 9). In summary, a pBUA-P_224_-based OMeY incorporation system was constructed and optimized for *B. subtilis*, which can efficiently incorporate OMeY into proteins at amber stop codons, reaching 58.50% of the gene expression level of non-engineered protein, with up to 24.82-fold activation under different OMeY concentrations.

### Balancing cell growth and NeuAc synthesis in *B. subtilis*

**Fig. 4:**
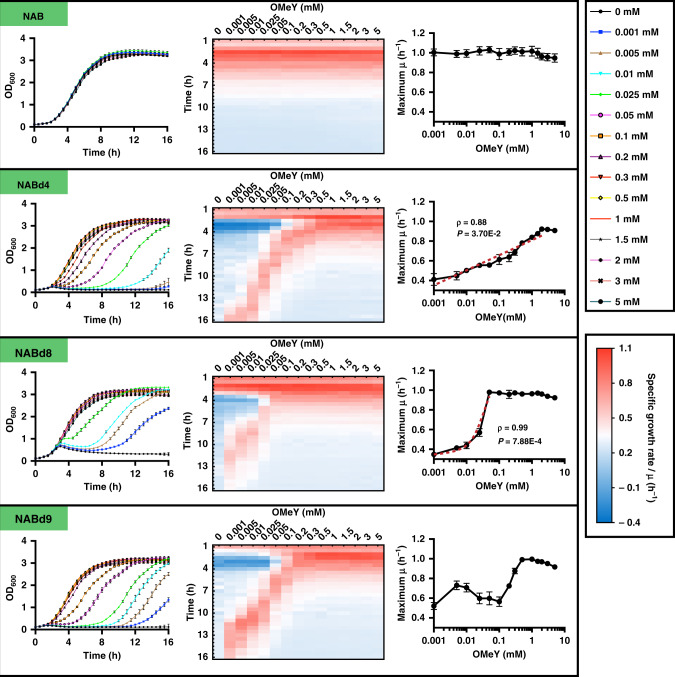
Effects of different *O*-methyl-L-tyrosine (OMeY) concentrations on the growth of OMeY-dependent, engineered *Bacillus subtilis* strains. The growth curves of the four engineered strains (left panels). Specific growth rates and maximum specific growth rates of the engineered strains at different OMeY concentrations are shown in the middle and right panels, respectively. *ρ*, Pearson correlations; *P*, *p*-value. All the data displayed as the mean ± SD (*n* = 4) of four biologically independent replicates. The statistical analysis was performed by one-sided *t*-test. Source data are provided as a Source data file.

*B. subtilis* has proven important for the bioproduction of vitamins, functional sugars, and industrial enzymes; however, cell growth often competes with target chemical biosynthesis for energy and precursors. Therefore, a robust cell growth and precise bioproduction regulation method is needed for *B. subtilis*. NeuAc biosynthesis involves not only competition for precursors of glycolysis and cell wall synthesis pathways, but also for phosphoenolpyruvate, a precursor of the TCA cycle. Therefore, NeuAc biosynthesis was selected as an example for *B. subtilis*, and the NeuAc biosynthetic strain with pBUA-P_224_, NAB, was constructed. Precisely controlling the expression level of essential genes by titrating OMeY should achieve precise regulation of cell growth, thereby fine-tuning the balance between cell growth and product synthesis. However, the regulation of the expression level of essential genes may affect endogenous metabolism of cells, and thus the generality of the GCE-CGBBE strategy. In order to minimize the impact on endogenous metabolism and identify metabolic pathway-independent target genes for cell growth control, ten genes related to cell wall synthesis and structural integrity were selected as candidates for the construction of OMeY-dependent *B. subtilis* growth. In order to minimize damage to protein function, the SIFT algorithm was used to predict TAG substitution sites^40^. To minimize the dosage of OMeY, the strong promoter P_43_ was used to replace endogenous promoters. In the end, three OMeY-dependent NeuAc producing strains were successfully constructed: NABd4, NABd8, and NABd9 by controlling the expression of *murB*, *walR*, and *cdsA*, respectively. The three gene encode UDP-*N*-acetylenolpyruvoylglucosamine reductase (peptidoglycan precursor biosynthesis), two-component response regulator (cell wall metabolism control) and phosphatidate cytidylyltransferase (phospholipids biosynthesis), respectively. Precise regulation of the expression levels of these three genes can theoretically slow cell division and further control cell growth rate. To test the effect of OMeY on growth regulation, the growth curves of three engineered and control strains were first determined at different concentrations of OMeY, and specific growth rates were calculated (Fig. 4). The growth of OMeY-dependent strains was able to respond to changes in OMeY concentration: (1) with an increase in OMeY concentration, the maximum specific growth rate of NABd4 increased logarithmically; (2) the maximum specific growth rate of NABd8 first increased linearly and then remained basically unchanged; (3) the maximum specific growth rate of NABd9 oscillated (Fig. 4). And after fusion of *murB*, *walR*, and *cdsA* with *gfp* gene, respectively, the expression levels of these three essential genes were also tested by monitoring fluorescence dynamics, which all increased first and then flattened with increasing OMeY concentration (Supplementary Fig. 10). Because GlcN6P is the precursor of cell wall peptidoglycan synthesis, the control of MurB expression in peptidoglycan synthesis pathway may affect metabolic flux to NeuAc biosynthetic pathway. Bioproduction and cell growth are difficult to be completely separated, therefore, the effects of both controlling cell growth and improving precursor GlcN6P supply may contribute to improved NeuAc production.

**Fig. 5:**
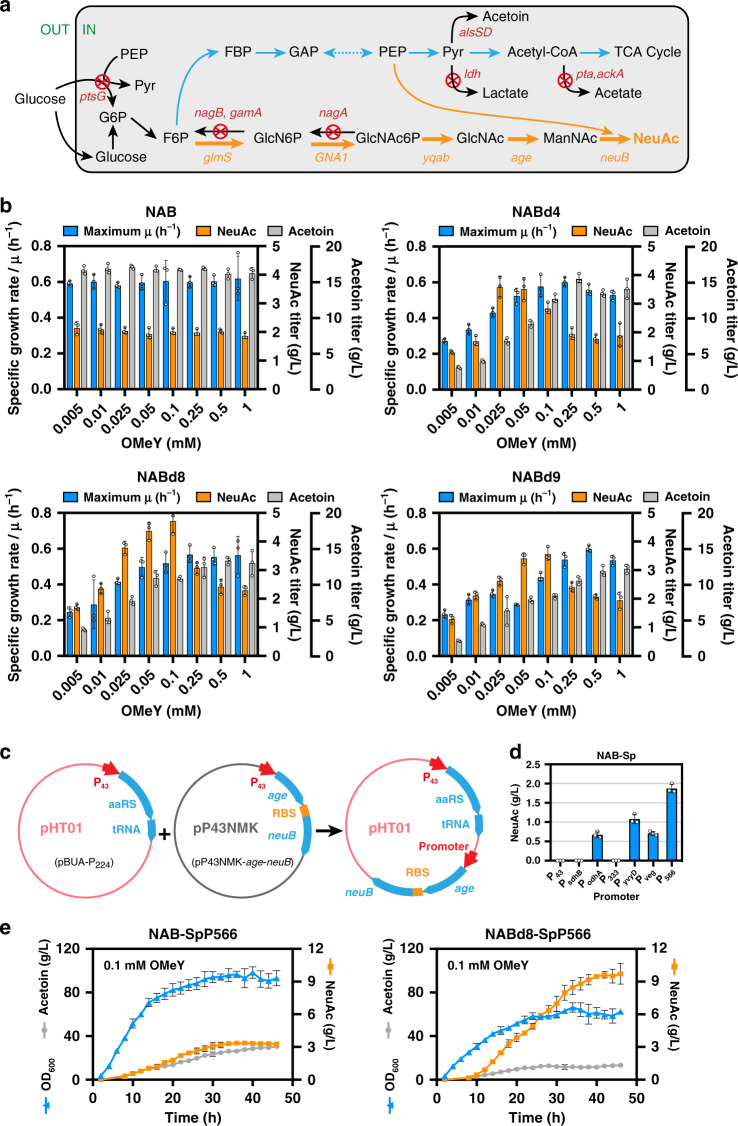
Balancing cell growth with biosynthesis of *N*-acetylneuraminic acid (NeuAc). **a** NeuAc biosynthetic pathways in *B. subtilis*. G6P, Glucose-6P; F6P, Fructose-6P; FBP, Fructose-1,6-2P; GAP, glyceraldehyde-3-P; PEP, phosphoenolpyruvate; GlcNAc, N-acetylglucosamine; ManNAc, N-acetylmannose. **b** Fermentation efficiencies of engineered *B. subtilis* strains at different *O*-methyl-L-tyrosine (OMeY) concentrations. **c** Amplifying gene *age* and *neuB* from plasmid pP43NMK and inserting into pBUA-P_224_ under the control of different promoters. **d** NeuAc production of seven single-plasmid NeuAc biosynthesis strains. **e** Fermentation efficiencies of NAB-SpP566 and NABd8-SpP566 in a 3-L fermenter. All the data are expressed as the mean ± SD from three (*n* = 3) biologically independent replicates. Source data underlying (**b**), (**d**), (**e**) are provided as a Source data file.

Fermentation tests on 24-well plates were carried out to verify a balance between cell growth and biosynthesis. By controlling essential genes using different concentrations of OMeY, the NeuAc titer of NABd8 increased to 2.34-fold, from 2.02 to 4.72 g/L, and from 0.13 to 0.34 g/L/OD_600_ (Fig. 5a, b; Supplementary Figs. 11, 12) when adding 0.1 mM OMeY. Besides, the molar ratio of NeuAc to by-product acetoin increased up to 5.12-fold from 0.034 to 0.174 mol/mol after growth control. When the titer of NeuAc reached its maximum level, the molar ratio of NeuAc to acetoin increased to 3.65-fold, reaching 0.124 mol/mol (Fig. 5b).

In order to test the effectiveness and robustness of the system at larger scale, we first constructed a single-plasmid strain by grafting gene *age* and *neuB* from plasmid pP43NMK to pBUA-P_224_ (Fig. 5c)^41^. At the same time, seven different promoters were tested to express gene *age* and *neuB* on pBUA-P_224_. Finally, a single-plasmid strain named NAB-SpP566 was obtained, which produced 1.87 g/L NeuAc (the strain harboring two plasmids, NAB, produced 2.02 g/L NeuAc) in 24-well plate (Fig. 5d). Then the strain NABd8-SpP566 was constructed by P_43_ promoter substitution and TAG codon incorporation for essential gene *walR* on the basis of NAB-SpP566. Subsequently, fed-batch fermentation was performed in a 3-L fermenter for strain NABd8-SpP566 with NAB-SpP566 as control. The NeuAc titer of NABd8-SpP566 reached 9.71 g/L, which was 2.97-fold of NAB-SpP566 (3.27 g/L). Moreover, NeuAc production of NABd8-SpP566 on cell reached 0.155 g/L/OD_600_, which was 4.43-fold of NAB-SpP566 (0.035 g/L/OD_600_, Fig. 5e). And the production of acetoin by-product decreased 56.42%, from 30.47 to 13.28 g/L. The above results preliminarily indicate the universality and stability of the GCE-CGBBE strategy in different strains using ncAA-incorporation tools.

### Biocontainment of OMeY-dependent *B. subtilis* strains

**Fig. 6:**
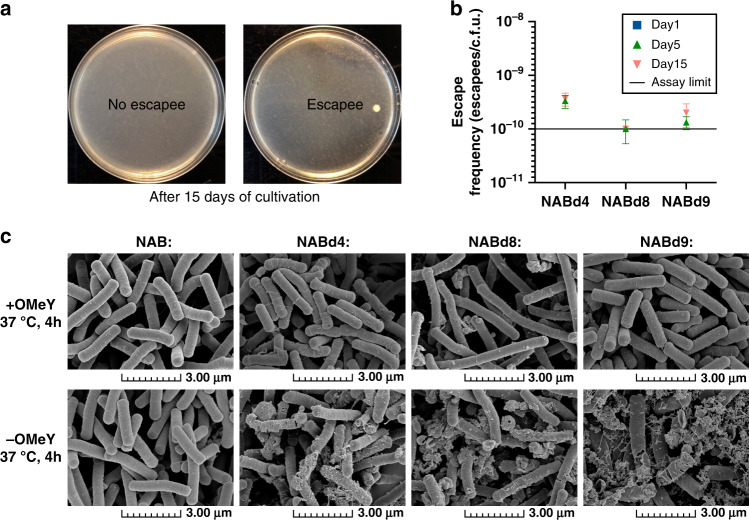
Determination of escape frequencies of *O*-methyl-L-tyrosine (OMeY)-dependent, engineered *Bacillus subtilis*. **a** After 15 days of cultivation, an escapee appeared on OMeY-free plates. **b** Escape frequencies of three OMeY-dependent *B. subtilis* strains were determined by plate coating. The escape efficiencies of all three strains on day 1 were under the assay limit, which is not shown in the plot. The data are expressed as the mean ± SEM from three (*n* = 3) biologically independent replicates. Source data are provided as a Source data file. **c** Scanning electron microscopic morphological identification of four engineered strains in the presence or absence of OMeY. Scanning electron microscopy experiments were performed three times independently with similar results. Source data underlying **b** are provided as a Source data file.

Unintended proliferation of genetically modified organisms (GMOs) is one of the major concerns for the biosafety of metabolic engineering research and industrial applications, which may pose potential threats to the survival of environmental microorganisms, plant growth, human and animal health, and may cause gene leakage by horizontal gene transfer^42–44^. Therefore, it is necessary to carry out biological containment of engineered microorganisms. For genetically recoded *E. coli* ∆321AM possessing an altered genetic code, ncAA-dependent strains have been constructed by inserting an amber stop codon to computationally redesigned essential genes to achieve biological containment^45,46^. From our design perspective, construction of the above OMeY-dependent *B. subtilis* cannot only achieve precise regulation of cell growth, but theoretically achieve the goal of biocontainment. Therefore, we tested the escape frequencies of NABd4, NABd8, and NABd9. The different strains were first cultured to mid-log phase in presence of 1 mM OMeY, washed, and subsequently incubated without OMeY to remove excess OMeY inside and outside the cells. By plating on OMeY-free plates, we found that the 15-day escape frequencies of all OMeY-dependent *B. subtilis* were lower than 3.67 × 10^−10^ escapees/c.f.u., meeting the requirement of 10^−8^ recommended by the National Institute of Health (Fig. 6a, b)^47^. These results demonstrate the effectiveness of ncAA-incorporation tools in biocontainment of non-genetically recoded industrial strains.

Due to the regulation of cell wall synthesis genes, cell morphology may change and further lead to a decline of production performance during large-scale fermentation. In order to test whether OMeY-dependent *B. subtilis* cell morphology was normal and whether the cell wall was damaged, cellular morphology was examined. In the presence of OMeY, the cell morphology was essentially the same as that of the control strain, NAB, except that NABd8 cells were longer than the control strain. In the absence of OMeY, the cell wall was ruptured, accompanied by content leakage (Fig. 6c). This provides evidence for the industrial application potential of ncAA-dependent strains, while at the same time highlighting their applicability in artificially controlled environments only.

## Discussion

Fine-tuning cell growth and biosynthesis are important for efficient bioproduction. To precisely balance these processes, a GCE-based orthogonal protein translation system was introduced into genetically recoded *E. coli* and non-genome-recoded *B. subtilis* to incorporate ncAA and further regulate metabolic flux and cell growth determinant genes by amber stop codon insertion. Proof-of-concept applications of metabolic engineering were verified by increasing GlcNAc and NeuAc in *E. coli* and *B. subtilis*, respectively. Thus, we demonstrated the potential of the combination of GCE and genetically recoded bacteria for precise regulation of cellular metabolism. Moreover, our highly efficient ncAA OMeY incorporation tool (pBUA), the first ever developed for *B. subtilis*, expanded ncAA-incorporated protein production to *B. subtilis*, which will potentially prove useful due to its strong protein secretion ability^48^. Given that the GCE-CGBBE strategy is pathway-independent and inactive without ncAAs that do not naturally occur in natural growth environments, it should hold generic and robust features for other important biochemical production in engineered bacteria.

Because only trace amounts of ncAAs need to be added to balance cell growth and bioproduction, GCE-CGBBE should be economically feasible. We calculated the costs of two selected ncAAs for large-scale fermentation. For pAcF, it would cost ~$14 per ton of fermentation broth at an optimal initial concentration of 0.01 mM. In parallel, for OMeY, it would cost ~$27 per ton of fermentation broth with an optimal initial concentration of 0.1 mM (Supplementary Table 1). These costs should be acceptable for bioproducing high value-added compounds. Furthermore, addition of ncAAs abolishes the need for monitoring glucose concentration, thereby simplifying the fermentation process, and reducing human labor. Moreover, based on the explosive growth of DNA sequencing throughput, and the availability of powerful bioinformatics tools, biosynthetic pathways for ncAAs from cheap precursors have been explored to produce at least 700 ncAAs, which promisingly further reduces ncAA cost^49^.

Potential applications of GCE-based ncAA-dependent strains are optimization processes for improving ratios of modular co-cultures in bioproduction, which is a promising strategy for overcoming unavailability of machineries and resources in individual cells by distributing precursor biosynthesis modules to multiple strains^50,51^. Currently, the control of the ratio of certain strains during modular co-culture relies on inoculation optimization and quorum-sensing system-based growth-regulation circuits^52,53^. A strategy for controlling this can be theoretically achieved in co-culture fermentation by titrating different ncAAs by constructing multiple different ncAA-dependent sub-populations.

Overall, the GCE-CGBBE strategy was developed as a generic approach for balancing bacterial growth and chemical biosynthesis for efficient bioproduction. Proof-of-concept applications for metabolic engineering revealed increased GlcNAc and NeuAc production in the best-studied Gram-negative (*E. coli*) and Gram-positive (*B. subtilis*) bacteria, yielding 4.54-fold and 2.34-fold increased titers, respectively. In addition, the developed ncAA-dependent essential gene expression regulation shows efficient biocontainment of engineered *B. subtilis*, which can prevent its unintended proliferation during metabolic research and industrial application. Because the GCE-CGBBE strategy is pathway-independent and does not interfere with ncAAs that do not naturally occur in any medium or growth environment, it should hold generic and robust features for additional important biochemical production in engineered bacteria.

## Methods

### Plasmids and strains

Gibson assembly was used to construct all plasmids used in this study with pET28a, pHT01, and p43NMK as backbones^54–57^. For genome editing of *E. coli* ∆321AM, λ-Red recombination was used, and pCP20 served to eliminate resistance selection markers on the *E. coli* genome. A homologous recombination box with 1000 bp homology arms was used to perform genome editing in *B. subtilis*, and the Cre-lox system was used to eliminate resistance selection markers on the genome.

Construction of the three pBUA libraries was as follows: based on pBUA-P1, we first selected 10- and 20-base sequences upstream of the mutant tRNA sequence, and replaced them with sequences of the same length composed of A, T, C, and G in equal probabilities to construct two random mutation libraries, named pBUA-10N and pBUA-20N, via degenerate primer design and PCR. Next, we mutated the 15-base DNA sequence between the −35 and −10 regions into a 15-base random sequence to construct the pBUA-15N library. For each mutant library, more than 6,000,000 cells were screened using flow cytometry, and ~1000 transformants with the highest single-cell fluorescence intensities were selected.

Construction of OMeY-dependent *B. subtilis*: First, a homologous recombination cassette with 1000 bp left homology arm, spectinomycin resistance cassette, P_43_ promoter and 1000 bp right homology arm (coding sequence of essential genes incorporated with TAG) was constructed by fusion PCR (for example, Seq S9-S11). Then the homologous recombination cassette was transformed into a strain harboring pBUA-P_224_ and the bacterial suspension was plated on a plate containing 1 mM OMeY and 100 mg/L spectinomycin. After 16–24 h plate incubation, all colonies were further screened with OMeY-free LB medium, in which OMeY-dependent *B. subtilis* could not grow successfully. And if there are no colonies on the plate or no positive transformants are obtained after three experiments, it is considered that the essential genes cannot be used to construct OMeY-dependent strains.

All plasmids and strains used in this study are shown in Supplementary Data 1. Snapgene 4.3.6 was used for gene sequence analysis and primer design, and the list of all primers is Supplementary Data 2. All sequences of genes, constructed plasmids and gene expression cassettes used in this study are provided as Supplementary Data 3.

### Strains cultivation

For cell culture, Luria–Bertani (LB) medium (10 g/L tryptone, 5 g/L yeast extract, and 10 g/L NaCl) was used unless noted otherwise. To produce GlcNAc by fermentation in *E. coli*, TMM (TB-added minimal medium: 30 g/L glucose, 2.4 g/L tryptone, 4.8 g/L yeast extract, 1 g/L glycerol, 5.8 g/L KH_2_PO_4_, 3.3 g/L K_2_HPO_4_·3H_2_O, 3.55 g/L citric acid·H_2_O, 2.5 g/L MgSO_4_·7H_2_O, 5 g/L (NH_4_)_2_SO_4_, 6 g/L urea, 0.025 g/L CaCl_2_·H_2_O, 0.1 mg/L CoCl_2_·H_2_O, 0.1 mg/L CuSO_4_·5H_2_O, 5 mg/L FeSO_4_·7H_2_O, 0.33 mg/L MnSO_4_·H_2_O, 3.8 mg/L ZnSO_4_·7H_2_O, pH 7.0, adjusted using NH_4_OH. To produce NeuAc by fermentation in *B. subtilis*, BFM (*B. subtilis* fermentation medium: 12 g/L yeast extract, 6 g/L tryptone, 6 g/L (NH_4_)_2_SO_4_, 12.5 g/L K_2_HPO_4_·4H_2_O, 2.5 g/L KH_2_PO_4_, 3 g/L MgSO_4_·7H_2_O, and 60 g/L glucose, pH 7.0, adjusted using NH_4_OH.

Cell culture using 96-well plates: first, 96-well deep-well plate with 300 μL fresh LB medium per well, which were then cultured for 10 h at 37 °C with shaking at 750 rpm. Next, 10 µL of the culture were inoculated in a 96-well microtiter plate containing 190 µL fresh LB medium with different concentrations of ncAA (pAcF or OMeY) and incubated at 37 °C with shaking at 750 rpm until OD_600_ reached 1. Subsequently, 10 μL of the culture were similarly inoculated into 96-well black plates (Corning 3603) containing 190 μL fresh LB medium, cultured at 37 °C with shaking at 750 rpm, and fluorescence intensities and cell growth curves were determined.

For determining the growth curves of OMeY-dependent *B. subtilis* at different OMeY concentrations, a 96-well plate was used to reduce the effect of the sampling process on cell growth. OMeY-dependent *B. subtilis* were first cultured in fresh LB medium supplemented with 1 mM OMeY for 10 h at 37 °C and 220 rpm. Before inoculation with different concentrations of OMeY, the bacterial suspension was washed to reduce residual OMeY inside and outside the cells. Then the bacterial suspension was first centrifuged at 5000 × g for 3 min and then resuspended in fresh OMeY-free LB medium. After performing this step twice, the bacterial suspension was incubated for 1 h at 37 °C with shaking at 220 rpm. Next, the bacterial suspension was centrifuged again at 5000 × *g* for 3 min, and resuspended in fresh LB medium. Other operations and detection methods were the same as those used for culturing cells in a 96-well plate.

Fermentation experiments performed in 24-well deep-well plates: For *E. coli*, the colonies on the plates were first picked into 3 mL fresh LB medium and cultured for 9 h at 37 °C and 220 rpm. Then, 15 μL of bacterial suspension was inoculated into a 24-well deep-well plate containing 1.5 mL of TMM medium per well, and fermentation was performed at 37 °C and 220 rpm for 33 h. For OMeY-dependent *B. subtilis*, the colonies on the plates were first picked into 1.5 mL fresh BFM medium with 1 mM OMeY. Before inoculation, the bacterial suspension was washed to remove additional OMeY; the culture was centrifuged at 5000 × *g* for 3 min and resuspended in 3 mL fresh BFM medium, which was repeated twice. Thereafter, 15 μL of bacterial suspension was inoculated into a 24-well deep-well plate containing 1.5 mL of fermentation medium per well, and fermentation was performed at 37 °C and 220 rpm for 60 h. A breathable sterile film was placed on the 24-well plate to ensure sufficient oxygen supply. At 30 h of fermentation, 1.5 mL of the culture was concentrated to ~1.3 mL, and at 60 h of fermentation, 1.5 mL of the culture was concentrated to ~1.1 mL.

Fed-batch fermentation in 3-L fermenter: The fermentation medium consists of 24 g/L yeast extract, 24 g/L tryptone, 8 g/L (NH_4_)_2_SO_4_, 13 g/L K_2_HPO_4_·4H_2_O, 3 g/L KH_2_PO_4_, 3.5 g/L MgSO_4_·7H_2_O, 2 g/L NaCl, 12 g/L urea, and 30 g/L initial glucose. First, colonies were picked from the plates and inoculated into baffled 500-mL shake flasks containing 50 ml of BFM medium, which was then cultured at 37 °C with shaking at 220 rpm until OD_600_ reached 15. Next, 100 mL of the seed was inoculated into a 3-L fermenter (T&J-Atype; T&J Bio-engineering Co., Ltd, Shanghai, China) with an initial 1.8 L of fermentation medium. Keep pH at 7.1 using 15% NH_3_ and maintain glucose concentration within 10–30 g/L by feeding with 700 g/L glucose throughout the fermentation period. The temperature was maintained at 37 °C, the aeration rate was maintained at 1.5 v.v.m. and the agitation speed was maintained at 800 rpm. Sampling every 2 h after inoculation to determine OD_600_, NeuAc titer, by-product acetoin titer and glucose concentration.

### Essential gene selection and TAG codon incorporation

Selection of essential genes for *B. subtilis* was based on three databases^58–60^. In order to avoid an influence of the essential gene expression change on target metabolites, ten essential genes related to cell wall synthesis were selected: *tagO*, *ftsW*, *ftsZ*, *murB*, *murC*, *groes*, *rodA*, *walR*, *cdsA*, and *yerQ*.

For the selection of TAG integration sites, the SIFT algorithm was used to predict residue tolerance of the proteins (http://sift.jcvi.org)^40^. The SIFT algorithm can predict amino acid substitutions that have little effect on protein function by using sequence homology. Of all the tolerable residue sites, we selected those sites closest to the translation start codon (ATG) to facilitate genome editing. However, if the distance between all predicted tolerable residue sites and the translation start codon was longer than 45 bases, and the active site of the protein was not at the N-terminus, the amber stop codon was inserted next to the translation start codon, which is the simplest method to prevent the production of truncated proteins^32,45^. The ten selected essential genes in the genome underwent P_43_ promoter (strong) substitution and TAG codon incorporation, respectively, by homologous recombination. Finally, three OMeY-dependent strains (based on three essential genes, *murB*, *walR*, and *cdsA*) were successfully constructed and sequenced: NABd4, NABd8, and NABd9 (Supplementary Data 1).

### Analytical methods

Cell mass was determined by measuring OD_600_. Fluorescence intensities were measured using a Cytation 3 Multi-Mode Reader (BIOTEK) at excitation and emission wavelengths of 488 and 523 nm, respectively. Gen5 CHS 2.06 was used to collect the biomass and fluorescence intensity data. While measuring the fluorescence intensities (FLS) and OD_600_ (ODS) of the samples, fluorescence intensity (FLB) and OD_600_ (ODB) of the blank medium were measured for fluorescence intensity correction. The final fluorescence intensity per unit biomass (FL/OD_600_) was calculated as (FLS-FLB)/(ODS-ODB).

High-performance liquid chromatography (HPLC, Agilent 1200 Series, USA) with an ultraviolet absorption detector (210 nm) was used to detect the concentrations of GlcNAc, NeuAc, and acetate in each sample. An Aminex HPX-87H column (300 × 7.8 mm, Bio-Rad, Hercules, CA, USA) was utilized, and the mobile phase was 10 mM H_2_SO_4_ with a flow rate of 0.5 mL/min at 40 °C. Agilent OpenLAB Control Panel was used to collect the HPLC data.

The glucose concentration during fermentation was measured using a glucose-lactate analyzer (M100, Shenzhen Sieman Technology Co., Ltd, Shenzhen, China). Microsoft Excel 2019 16.37 was used to analyze the biomass, yield and fluorescence intensity data, and carry out statistical calculation. Fluorescent-activated cell sorting (FACS) technology was used to screen single cells with high fluorescence intensities. Cell analyzer (BD FACSAria III, BD, Franklin Lakes, NJ, USA) with FlowJo_V10 (FlowJo, LLC) software was used for cell sorting. For each random mutation library, 6,000,000 transformants were determined and screened to obtain ~1000 potential target transformants.

### Model simulation

MATLAB R2019a was used for kinetic modeling of cell growth, as well as for target metabolite (GlcNAc) and by-product (acetate) synthesis^61–63^. The development of the kinetic model was mainly based on four kinetics, including the growth kinetic model of acetic acid-producing *E. coli* developed by Luli et al., Monod equation describing nutrient limitation, acetate synthesis and specific growth rate equations determined by Basan et al., and Michaelis–Menten equation describing the biosynthesis pathway of GlcNAc^61,64,65^. This model mainly captures three characteristics of engineered *E. coli* in fermentation experiments: (1) addition of pAcF can change the specific growth rate by regulating the flux of the glycolytic pathway; (2) accumulation of acetate can inhibit both cell growth and biosynthesis of target metabolites and acetate; and (3) cell growth is also limited by the concentration of nutrients (especially nitrogen sources) in the medium. Besides, concentrations of metabolites and all parameters in the model are expressed in arbitrary units (a.u.) which is sufficient to qualitatively simulate the dynamic characteristics of metabolites and biomass. Based on these points, model construction and parameter selection were described as stated in the following sections.

First, according to the experimental results regarding the regulation of pAcF concentration on gene expression, the flux rate of glycolytic pathway (*v*_EMP_) per unit biomass per unit time was calculated as a function of the pAcF concentration (*x*_pacf_):

1 $$v_{{\mathrm{EMP}}} = A \cdot {\mathrm{ln}}(x_{{\mathrm{pacf}}}) + B$$

where *x*_pacf_ ranged from 0.1 to 10, and *v*_EMP_ ranged from 0.3 to 1 according to the experimental results of Hollinshead et al. regarding the culture test of *pfkA-*deficient *E. coli*^66^. Therefore, the parameters in the kinetic model were calculated as *A* = 0.152 and *B* = 0.65. According to the experimental results of Basan et al., as the glycolytic pathway flux rate (*v*_EMP_) gradually increases, the specific growth rate (*μ*) subsequently increases. Further, as the glycolytic pathway flux rate (*v*_EMP_) continues to increase, ‘overflow metabolism’ activates the acetate synthesis pathway, and *μ* increases linearly with the increase of acetate synthesis rate (*v*_ace_)^65^. Therefore, the promotion of the glycolytic pathway flux rate (*v*_EMP_) to *μ* is divided into two phases, including non-acetate-producing (*v*_EMP_ = 0.3–0.6) and acetate-producing (*v*_EMP_ = 0.6–1) phases. The *μ* of the intersection of the two phases (*μ*_0_ was set to 0.76 according to the experimental data of Basan et al., 0.76 h^−1^, to match the further parameters of cell growth) is taken as the rate of growth in the absence of all limitations. Therefore, in the model described, ‘*I*_EMP_’ is used as a coefficient to calculate the effect of glycolytic pathway flux rate on cell growth rate. During the non-acetate-producing phase, *I*_EMP_ (0.3–1) increases linearly with increasing *v*_EMP_. During the acetate-producing stage, the synthesis rate of acetate (*v*_ace_ was ranged from 0 to 0.24 according to the experimental data of Basan et al., 0–0.24 g/OD_600_/h) increases linearly with increasing *v*_EMP_, while *I*_EMP_ (*I*_EMP_ was ranged from 1 to 1.6 to achieve an up to 1.6 times’ increase on *μ* according to the results of Basan et al.) increases linearly with increasing *v*_ace_^65,67^. Therefore, the relationship between *I*_EMP_ and *x*_pacf_ could be modeled by the following equation with parameters calculated as *C* = 7/3, *D* = −0.4, *E* = 1.5, and *F* = 0.1.

2 $$I_{{\mathrm{EMP}}} = \left\{ {\begin{array}{*{20}{c}} {C\cdot v_{{\mathrm{EMP}}} + D\;\;{\mathrm{for}}\;0.3 \le v_{{\mathrm{EMP}}} \le 0.6} \\ \!\!\!\!\!{E\cdot v_{{\mathrm{EMP}}} + F\;\;\,{\mathrm{for}}\;0.6 < v_{{\mathrm{EMP}}} \le 1} \end{array}} \right.$$

The acetate biosynthesis rate (*v*_ace_) could be modeled by the following equation: *G* = 0.6, *H* = −0.36.

3 $$v_{{\mathrm{ace}}} = \left\{ {\begin{array}{*{20}{c}} {0\;\;\;{\mathrm{for}}\;0.3 \le v_{{\mathrm{EMP}}} \le 0.6} \\ {G\cdot v_{{\mathrm{EMP}}} + H\;\;{\mathrm{for}}\;0.6 < v_{{\mathrm{EMP}}} \le 1} \end{array}} \right.$$

And the inhibition factor *I*_ace_ of acetate for cell growth was calculated according to the results of Luli et al.^64^:

4 $$I_{{\mathrm{ace}}} = \frac{{k_1}}{\mu }$$

where *k*_1_ was calculated as a function of *x*_ace_ according to the results of Pinhal et al., and *x*_ace_ has the following dynamic:

5 $$k_1 = 0.76\cdot 0.0534^{x_{{\mathrm{ace}}}} + 0.1$$

6 $$\frac{{{\mathrm{d}}x_{{\mathrm{ace}}}}}{{{\mathrm{d}}t}} = I_{{\mathrm{ace}}}\cdot v_{{\mathrm{ace}}}\cdot x_{{\mathrm{cell}}}$$

In addition to the effects of glycolytic flux rate and acetic acid accumulation, cell growth rate is also limited by nitrogen source (*x*_N_) under conditions of sufficient carbon source (glucose) in fermentation experiment. Therefore, we also set the coefficient ‘*I*_N_’ to calculate the effects of nutrients on cell growth rate according to Monod equation:

7 $$I_{\mathrm{N}} = \frac{{x_{\mathrm{N}}}}{{x_{\mathrm{N}} + K_{\mathrm{S}}}}$$

where *x*_N_ is the residual nitrogen source in the fermentation system, *x*_N0_ is the initial nitrogen source, and *K*_S_ is the half-saturation constant (*K*_S_ = 5, 10, 15, 20) of Monod equation (Supplementary Fig. 4). In the simulated fermentation system, the nitrogen source is used for conversion into biomass and GlcNAc, the theoretical conversion rates of which are *Y*_X/N_ = 0.7 and *Y*_GNA/N_ = 0.54, respectively. Therefore, *x*_N0_ could be modeled by the following equation:

8 $$x_{\mathrm{N}} = x_{{\mathrm{N}}0} - \frac{{x_{{\mathrm{cell}}}}}{{Y_{{\mathrm{X/N}}}}} - \frac{{x_{{\mathrm{GNA}}}}}{{Y_{{\mathrm{GNA}}/{\mathrm{N}}}}}$$

After calculating the three parameters, *I*_EMP_, *I*_ace_, and *I*_N_, which affect cell growth rate, the biomass (*x*_cell_) has the following dynamics:

9 $$\mu = I_{{\mathrm{EMP}}}\cdot I_{{\mathrm{ace}}}\cdot I_{\mathrm{N}}\cdot \mu _0$$

10 $$\frac{{dx_{{\mathrm{cell}}}}}{{{\mathrm{dt}}}} = x_{{\mathrm{cell}}}\cdot \mu$$

In addition, Michaelis–Menten equation was used for qualitative simulation of GlcNAc biosynthesis. All *K*_m_ values in the metabolic pathway were equal to 1, and all maximal reaction rates (*v*_max_) were 0.24, in order to match the synthesis rate of acetate. At the same time, the biosynthesis rate (*v*_GNA_) of GlcNAc was multiplied by *I*_ace_ to inhibit accumulation. Therefore, the GlcNAc concentration (*x*_GNA_) has the following dynamics:

11 $$v_{{\mathrm{GNA}}} = I_{{\mathrm{ace}}}\cdot \frac{{v_{{\mathrm{GNAmax}}} \cdot x_{{\mathrm{GlcNAc6P}}}}}{{x_{{\mathrm{GlcNAc6P}}} + K_{{\mathrm{m}}6}}}$$

12 $$ \frac{{{\mathrm{d}}x_{{\mathrm{GNA}}}}}{{{\mathrm{d}}t}} = x_{{\mathrm{cell}}}\cdot v_{{\mathrm{GNA}}}$$

where *v*_max_ of YqaB per unit biomass per unit time (*v*_GNAmax_) was 0.24, and *K*_m_ of YqaB (*K*_m6_) was 1. The initial parameters of the model are initial nitrogen source *x*_N0_ = 50 and initial biomass *x*_cell0_ = 0.1.

### Solid-media escape assays

Escape frequency was determined according to methods already reported in previous studies^46^. Briefly, the OMeY-dependent strain was first cultured in 50 mL of fresh LB medium containing 1 mM OMeY for 10 h at 37 °C and 220 rpm. Cells were then washed by centrifugation and resuspended in fresh LB medium. After the cells were washed twice, OD_600_ was measured. Three technical replicates were performed for each strain. For each sample, ~10^10^ cells (it is estimated that the bacterial suspension with an OD_600_ of 1 contains ~20,000,000 cells per mL) were plated onto OMeY-free, solid LB plates. Next, the numbers of escapees growing on the plates were counted after 1, 5, and 15 days of culture. At the same time, the bacterial suspension was diluted and plated on an LB plate with 1 mM OMeY to count the total number of cells. Escape frequencies for each OMeY-dependent strain were calculated as mean of escapees per colony forming unit (c.f.u.), plated on three OMeY-free LB solid plates. Standard error of the mean (SEM) was calculated as described by Mandell et al.^46^.

### Reporting summary

Further information on research design is available in the Nature Research Reporting Summary linked to this article.

## Supplementary information

Supplementary Information

Reporting Summary

Description of Additional Supplementary Files

Supplementary Data 1

Supplementary Data 2

Supplementary Data 3

## Data Availability

The authors declare that all data supporting the findings of this study are available within the paper and its Supplementary Information files. A reporting summary for this article is available as a Supplementary Information file. The datasets generated and analyzed during the current study are also available from the corresponding author upon request. Source data are provided with this paper.

## Source data

Source data

## Code availability

The MATLAB code used in this study can be found in GitHub [https://github.com/Tian-RZ/Eco_kinetic_model.git].
