# Supplementary material for: Titrating bacterial growth and chemical biosynthesis for efficient N-acetylglucosamine and N-acetylneuraminic acid bioproduction
Source: Nat Commun. 2020 Oct 8;11:5078. doi: 10.1038/s41467-020-18960-1 (PMC7544899; doi:10.1038/s41467-020-18960-1)
Supplement: Supplementary file 1 — Supplementary Information [file 41467_2020_18960_MOESM1_ESM.pdf]

Titrating bacterial growth and chemical biosynthesis for efficient *N*-acetylglucosamine and *N*-acetylneuraminic acid bioproduction

Tian *et al.*

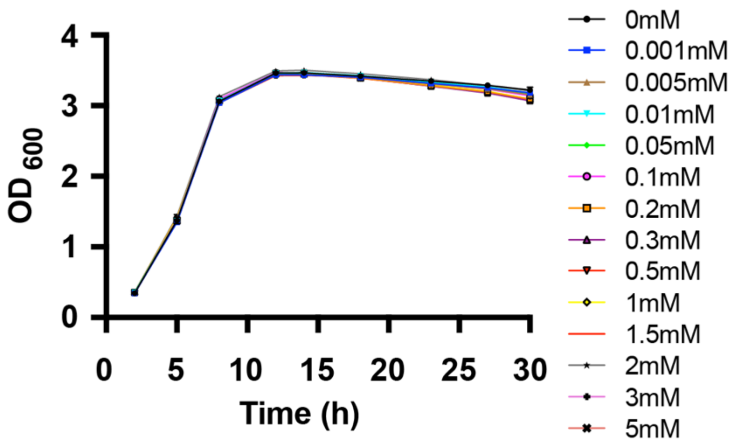

Supplementary Figure 1. Growth curve of *Escherichia coli* Δ321AM-pEVOL in LB medium with different pAcF concentrations. Optical density at 600 nm (OD_{600}) was used to characterize biomass and quintuplicate experiments were carried out for characterizing OD_{600} of *E. coli* Δ321AM-pEVOL at different times. The data are expressed as the mean ± SD from five (n = 5) biologically independent replicates. Source data are provided as a Source Data file.

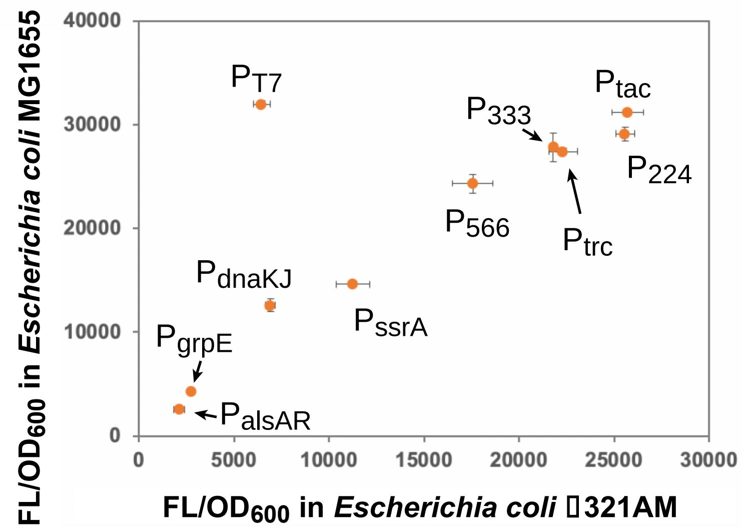

Supplementary Figure 2. A series of tested promoters and their relative strengths in *E. coli* MG1655 and *E. coli* 321. Green fluorescent protein (GFP) gene was used as a reporter gene. The data are expressed as the mean ± SD from five (n = 5) biologically independent replicates. Source data are provided as a Source Data file.

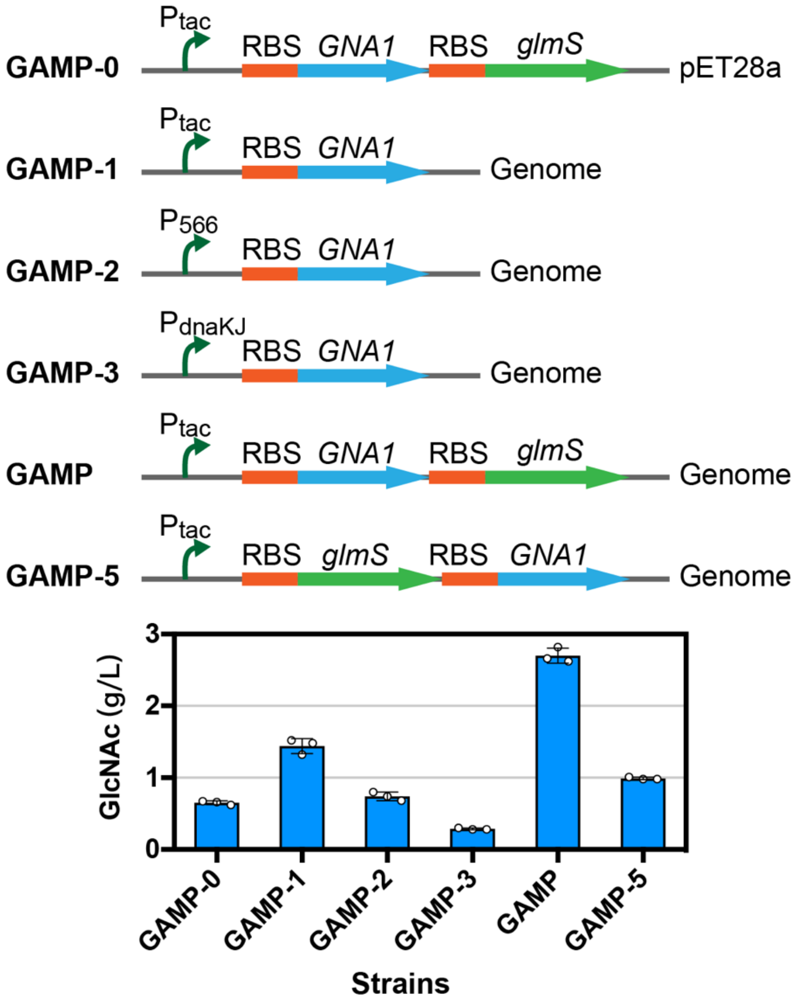

Supplementary Figure 3. Fermentation results of five engineered GlcNAc-producing strains using TMM medium. The data are expressed as the mean ± SD from three (n = 3) biologically independent replicates. Source data are provided as a Source Data file.

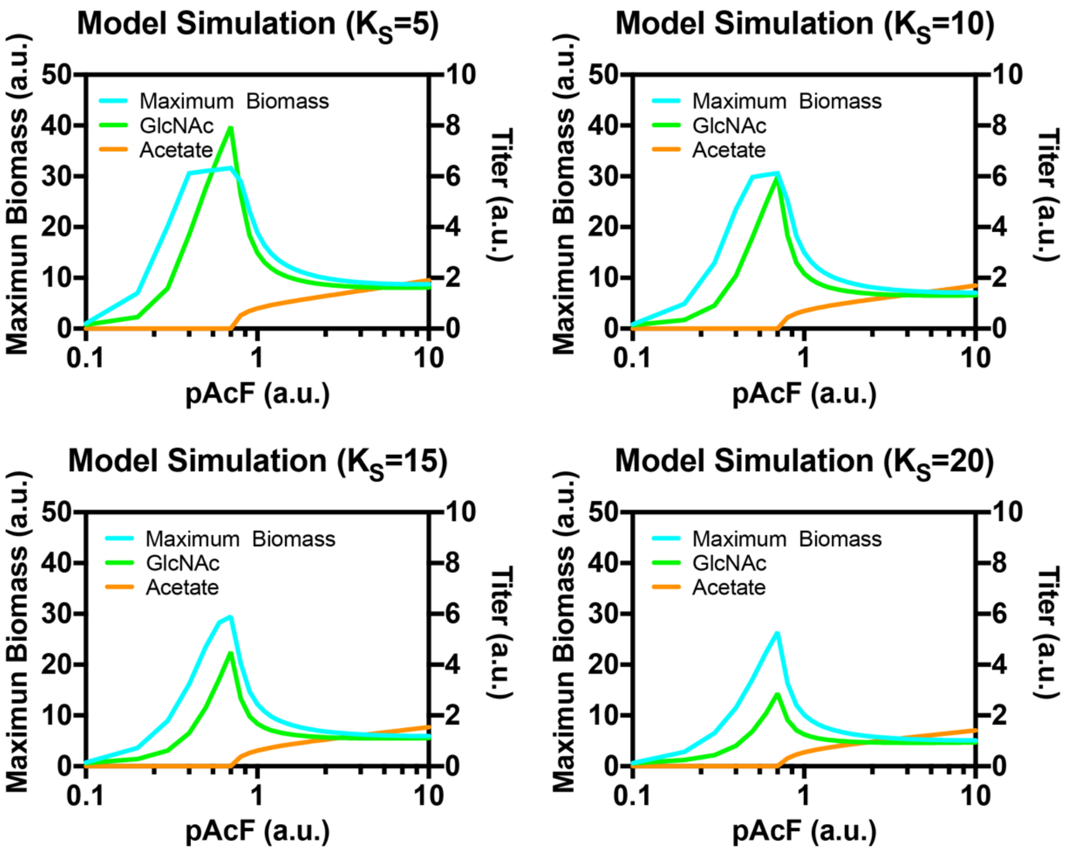

Supplementary Figure 4. The model simulated fermentation results. Source data are provided as a Source Data file.

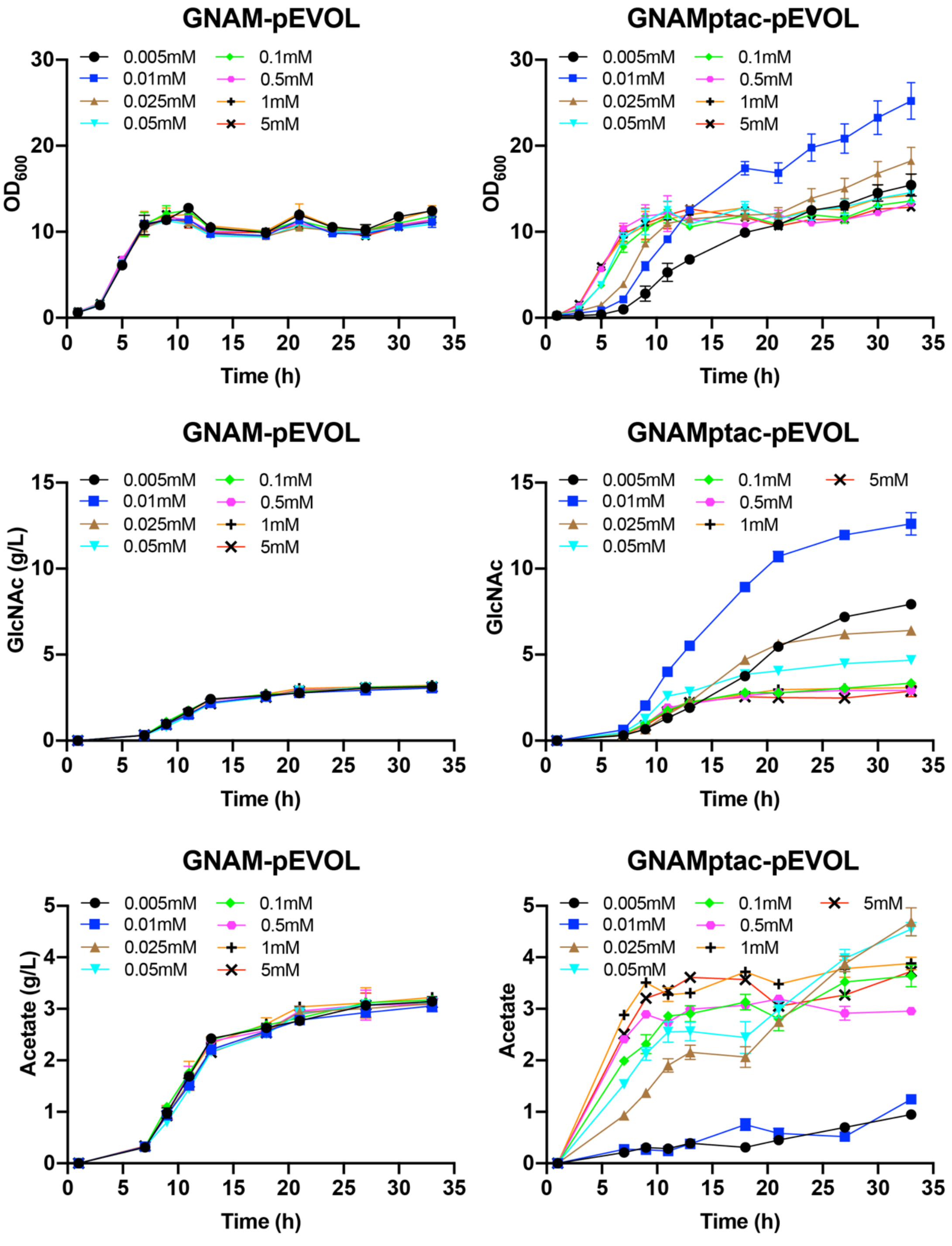

Supplementary Figure 5. 24-well plate fermentations of GNAM-pEVOL and GNAMptac-pEVOL using TMM medium with different pAcF concentrations. The data are expressed as the mean ± SD from three (n = 3) biologically independent replicates. Source data are provided as a Source Data file.

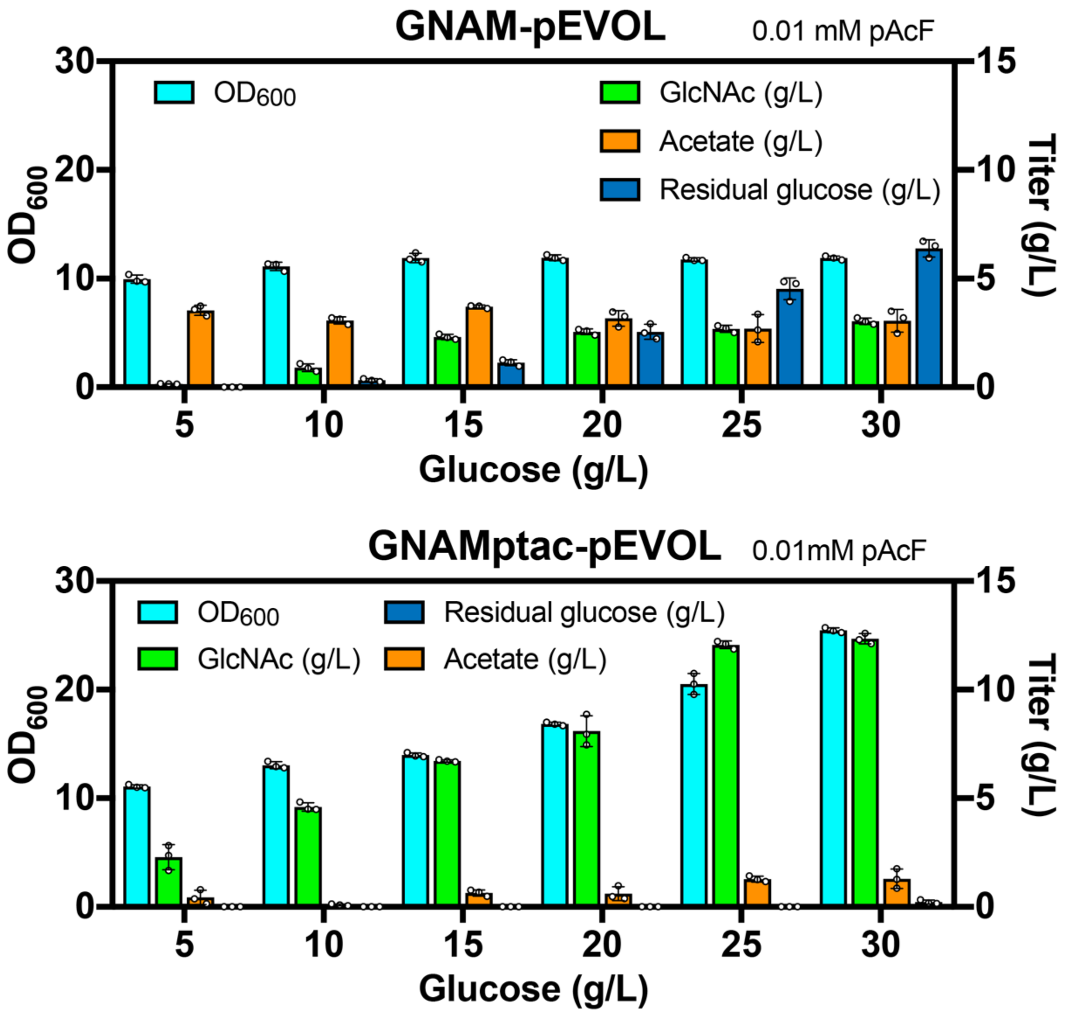

Supplementary Figure 6. Fermentation of GNAM-pEVOL and GNAMptac-pEVOL using TMM medium with different initial glucose concentrations. The data are expressed as the mean ± SD from three (n = 3) biologically independent replicates. Source data are provided as a Source Data file.

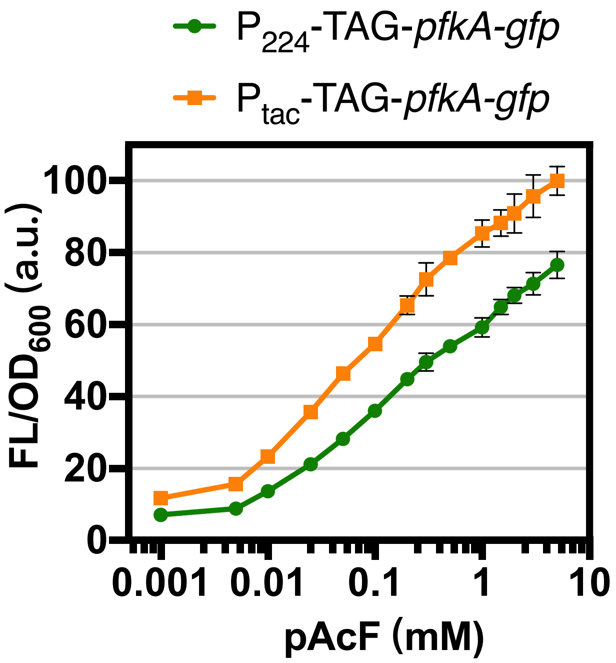

Supplementary Figure 7. *pfkA* expression levels at different concentrations of pAcF using GFP as a reporter. The data are expressed as the mean ± SD from three (n = 3) biologically independent replicates. Source data are provided as a Source Data file.

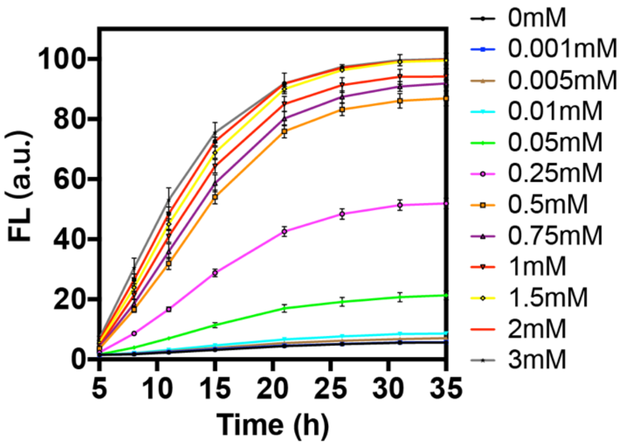

Supplementary Figure 8. Relative fluorescence intensity curve of BSU168-pBUA in LB medium with different OMeY concentrations. The data are expressed as the mean ± SD from five (n = 5) biologically independent replicates. Source data are provided as a Source Data file.

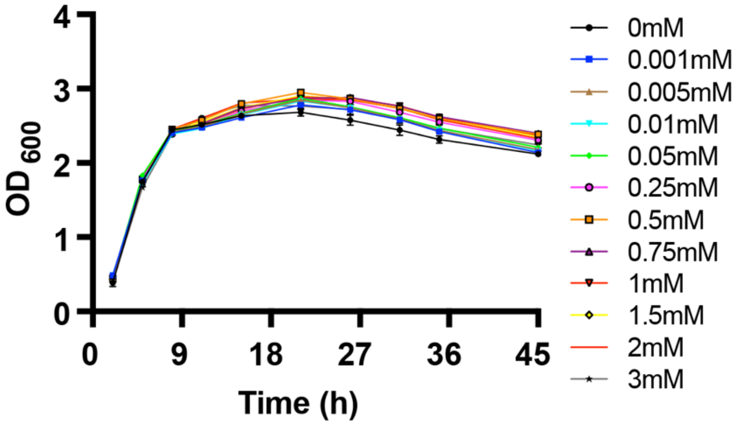

Supplementary Figure 9. Growth curve of BSU168-pBUA in LB medium with different OMeY concentrations. Optical density at 600 nm (OD_{600}) was used to characterize biomass. The data are expressed as the mean ± SD from five (n = 5) biologically independent replicates. Source data are provided as a Source Data file.

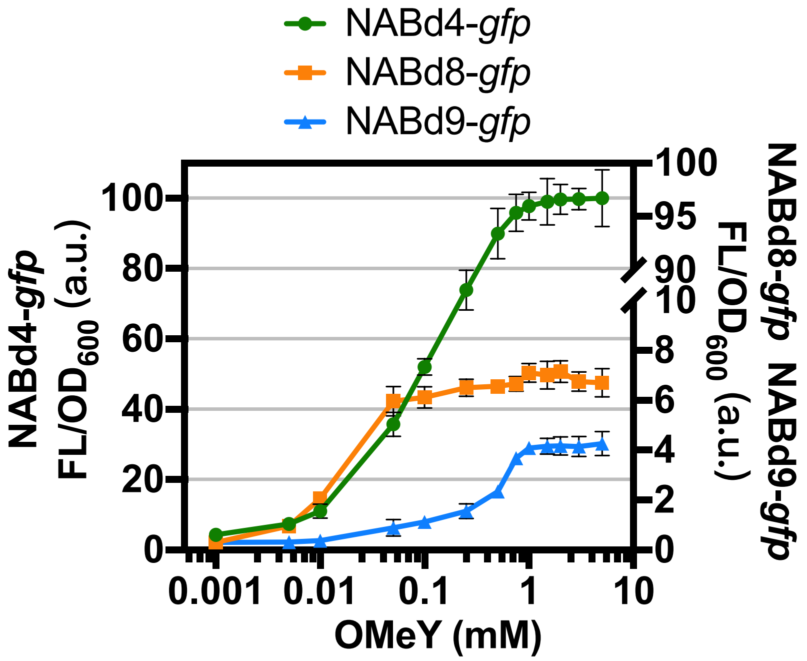

Supplementary Figure 10. Expression levels of three essential genes (*murB*, *walR*, *cdsA*) at different concentrations of OMeY using GFP as a reporter. The data are expressed as the mean ± SD from three (n = 3) biologically independent replicates. Source data are provided as a Source Data file.

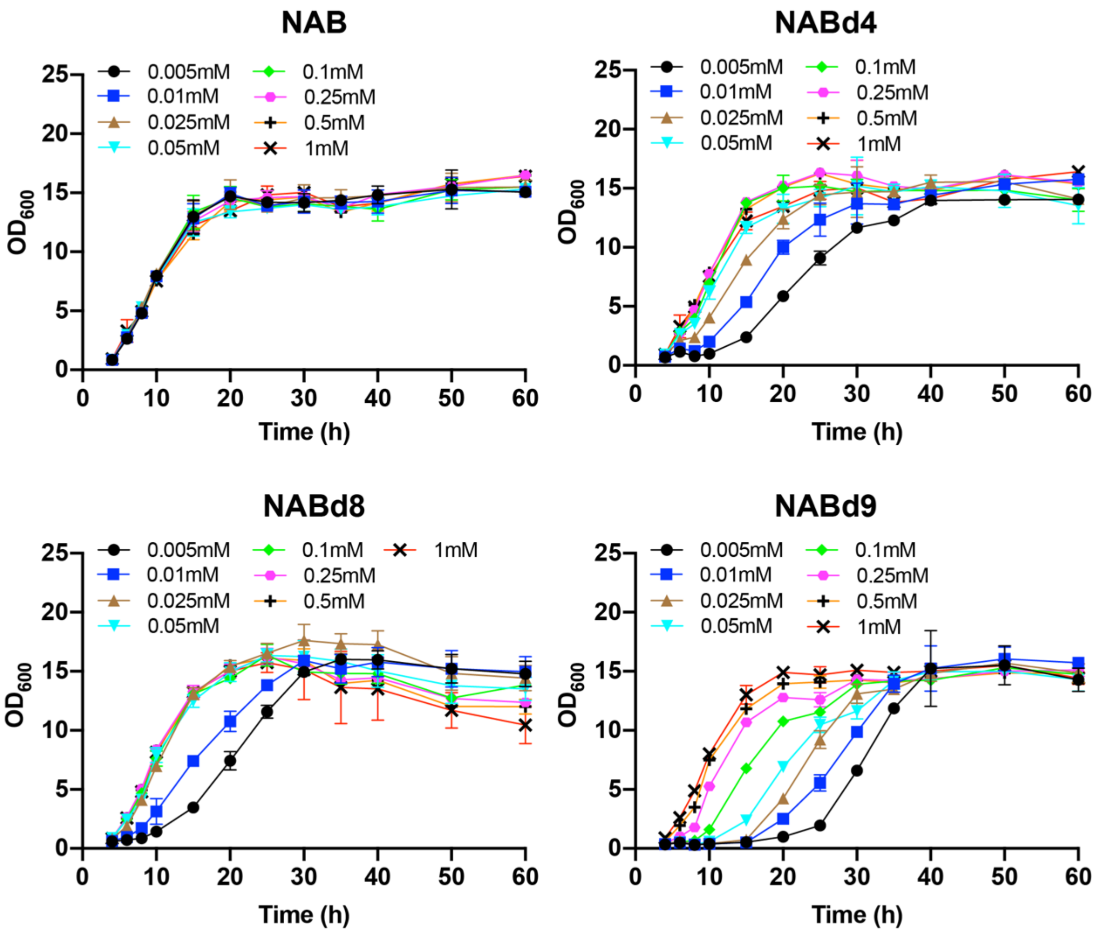

Supplementary Figure 11. Growth curve of four engineered *B. subtilis* fermentation experiments using BFM medium with different OMeY concentrations in 24-well plates. The data are expressed as the mean ± SD from three (n = 3) biologically independent replicates. Source data are provided as a Source Data file.

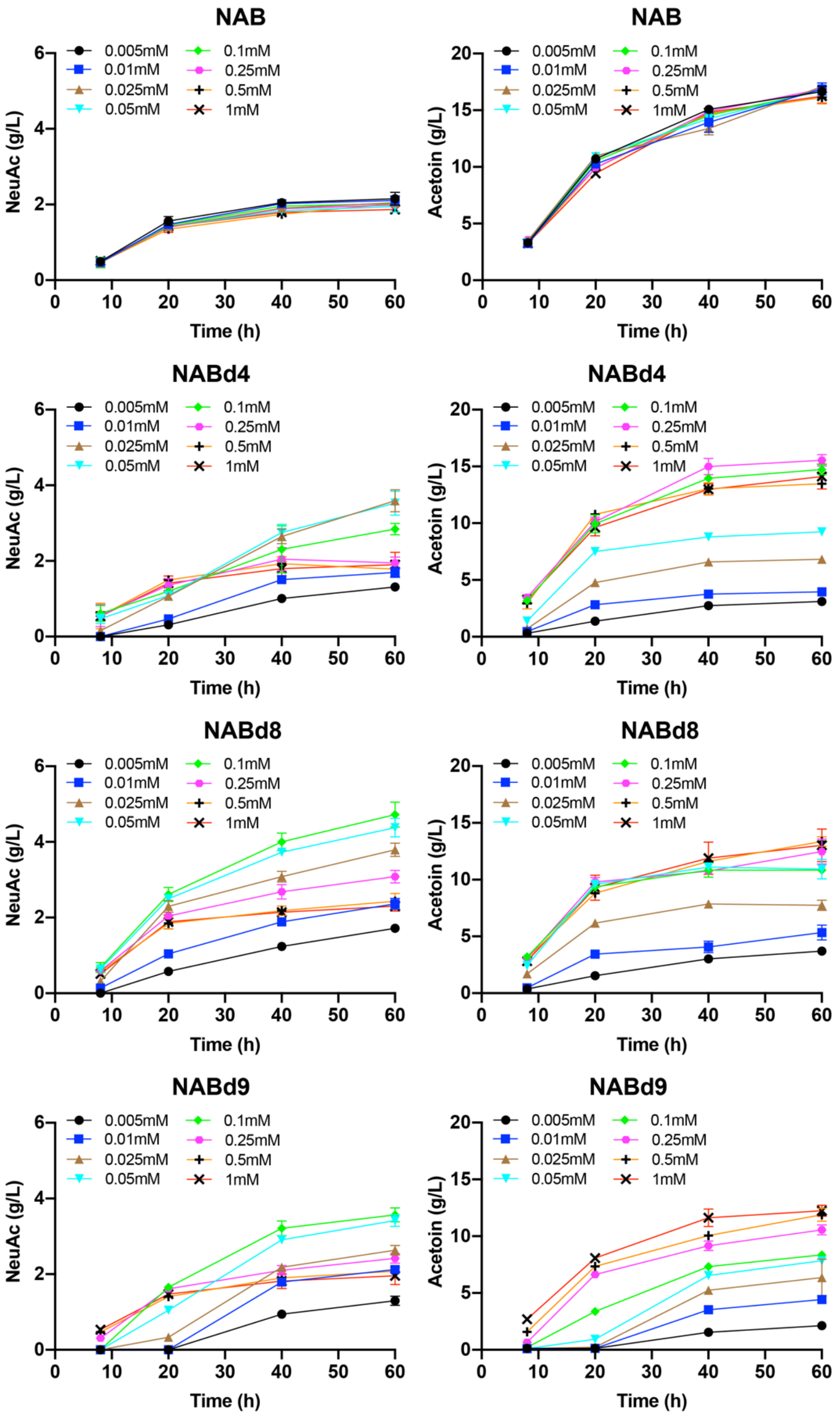

Supplementary Figure 12. 24-well plate fermentations of four engineered *B. subtilis* using BFM medium with different OMeY concentrations. The data are expressed as the mean ± SD from three (n = 3) biologically independent replicates. Source data are provided as a Source Data file.

Supplementary Table 1. Cost per cubic of culture for ncAAs used in this study.

Name of ncAA	CAS#	MW Cat# for 1g	Price of 1g ($)	Optimal conc. (mM)	Cost per cubic of culture ($)
4-Acetyl-L-phenylalanine (pAcF)	2018-61-3	207.23	6.8	0.01-0.025	14 -35
4-Methoxy-L-tyrosine (OMeY)	6230-11-1	195.22	1.4	0.1-0.5	27-75
