# Supplementary material for: Titrating bacterial growth and chemical biosynthesis for efficient N-acetylglucosamine and N-acetylneuraminic acid bioproduction
Source: Nat Commun. 2020 Oct 8;11:5078. doi: 10.1038/s41467-020-18960-1 (PMC7544899; doi:10.1038/s41467-020-18960-1)
Supplement: Supplementary file 2 — Reporting Summary [file 41467_2020_18960_MOESM2_ESM.pdf]

nature research

Corresponding author(s): Yanfeng Liu and Jian Chen

Last updated by author(s): Sep 13, 2020

Reporting Summary

Nature Research wishes to improve the reproducibility of the work that we publish. This form provides structure for consistency and transparency in reporting. For further information on Nature Research policies, see our Editorial Policies and the Editorial Policy Checklist.

Statistics

For all statistical analyses, confirm that the following items are present in the figure legend, table legend, main text, or Methods section.

n/a	Confirmed	
☐	☒	The exact sample size (*n*) for each experimental group/condition, given as a discrete number and unit of measurement
☐	☒	A statement on whether measurements were taken from distinct samples or whether the same sample was measured repeatedly
☐	☒	The statistical test(s) used AND whether they are one- or two-sided<br>*Only common tests should be described solely by name; describe more complex techniques in the Methods section.*
☒	☐	A description of all covariates tested
☒	☐	A description of any assumptions or corrections, such as tests of normality and adjustment for multiple comparisons
☐	☒	A full description of the statistical parameters including central tendency (e.g. means) or other basic estimates (e.g. regression coefficient) AND variation (e.g. standard deviation) or associated estimates of uncertainty (e.g. confidence intervals)
☐	☒	For null hypothesis testing, the test statistic (e.g. *F*, *t*, *r*) with confidence intervals, effect sizes, degrees of freedom and *P* value noted<br>*Give P values as exact values whenever suitable.*
☒	☐	For Bayesian analysis, information on the choice of priors and Markov chain Monte Carlo settings
☒	☐	For hierarchical and complex designs, identification of the appropriate level for tests and full reporting of outcomes
☒	☐	Estimates of effect sizes (e.g. Cohen's *d*, Pearson's *r*), indicating how they were calculated

Our web collection on statistics for biologists contains articles on many of the points above.

Software and code

Policy information about availability of computer code

Data collection	Agilent OpenLAB Control Panel was used to collect the HPLC data; Gen5 CHS 2.06 was used to collect the biomass and fluorescence intensity data; BD FACS AriaTM III apparatus cell analyzer with FlowJo_V10 (FlowJo, LLC) software was used for cell sorting.
Data analysis	Microsoft excel 2019 16.37 was used to analyze the biomass, yield and fluorescence intensity data, and carry out statistical calculation. Matlab R2019a are used to construct kinetic models. Snapgene 4.3.6 was used for gene sequence analysis and primer design. SIFT algorithm was used predict TAG substitution site.

For manuscripts utilizing custom algorithms or software that are central to the research but not yet described in published literature, software must be made available to editors and reviewers. We strongly encourage code deposition in a community repository (e.g. GitHub). See the Nature Research guidelines for submitting code & software for further information.

Data

Policy information about availability of data

All manuscripts must include a data availability statement. This statement should provide the following information, where applicable:

- Accession codes, unique identifiers, or web links for publicly available datasets
- A list of figures that have associated raw data
- A description of any restrictions on data availability

The authors declare that all data supporting the findings of this study are available within the paper and its supplementary information files. A reporting summary for this article is available as a Supplementary Information file. The datasets generated and analyzed for this study are also available from the corresponding author upon request. The source data underlying Figures 2a, 2d, 3a, 3c, 3d, 4, 5b, 5d, 5e, 6b and supplementary figures 1-12 are provided as a Source Data file. All the sequence data used in this article can be found in the supplementary information file. Source data are provided with this paper.

Field-specific reporting

Please select the one below that is the best fit for your research. If you are not sure, read the appropriate sections before making your selection.

- [x] Life sciences
- [ ] Behavioural & social sciences
- [ ] Ecological, evolutionary & environmental sciences

For a reference copy of the document with all sections, see nature.com/documents/nr-reporting-summary-flat.pdf

Life sciences study design

All studies must disclose on these points even when the disclosure is negative.

Sample size	No sample sized calculation was performed. This experiment was performed on bacteria cultivation in 24-well plate, 96-well plate or in 3-L fermenters. As indicated in the text, all experiments were performed from a single colony. Sample size was 3 or more for all plates fermentation, and 3 for fed-batch cultivation. For flow cytometry, we screened 6,000,000 cells/ sample; For solid-media escape assay, approximately 1,000,000,000 cells/ sample were plated onto a OMeY-free, solid LB plate.
Data exclusions	There is no data exclusion in our study.
Replication	At least two biologically independent replicates was performed for each experiment to ensure reliability. We have encountered no problems with reproducibility.
Randomization	The colonies used in all experiments were randomly picked from the plates.
Blinding	Blinding was not relevant to this study, because the analysis was carried out entirely on bacteria.

Reporting for specific materials, systems and methods

We require information from authors about some types of materials, experimental systems and methods used in many studies. Here, indicate whether each material, system or method listed is relevant to your study. If you are not sure if a list item applies to your research, read the appropriate section before selecting a response.

Materials & experimental systems

n/a	Involved in the study	
x		Antibodies
x		Eukaryotic cell lines
x		Palaeontology and archaeology
x		Animals and other organisms
x		Human research participants
x		Clinical data
x		Dual use research of concern

Methods

n/a	Involved in the study	
x		ChIP-seq
	x	Flow cytometry
x		MRI-based neuroimaging

Flow Cytometry

Plots

Confirm that:

- [x] The axis labels state the marker and fluorochrome used (e.g. CD4-FITC).
- [x] The axis scales are clearly visible. Include numbers along axes only for bottom left plot of group (a 'group' is an analysis of identical markers).
- [x] All plots are contour plots with outliers or pseudocolor plots.
- [x] A numerical value for number of cells or percentage (with statistics) is provided.

Methodology

Sample preparation	For a fluorescence assay, cells were washed twice and resuspended in PBS (phosphate-buffered solution) to an OD600 of 0.2.
Instrument	BD FACS AriaTM III apparatus cell analyzer (BD Biosciences).
Software	FlowJo_V10 (FlowJo, LLC)
Cell population abundance	About 6,000,000 cells were sorted for each sample.

Gating strategy

The assays were performed using a LSR FortessaTM LSRII cell analyzer (BD Biosciences) using FITC (GFP, green fluorescent protein) channels with a voltage gain of 407 V. Compensation was performed using cells that harboring a GFP gene without TAG substitution. For each sample, about 6,000,000 counts were sorted using a 0.5 mL· s-1 flow rate. A gate was previously designed based on forward and side scatter.

☒ Tick this box to confirm that a figure exemplifying the gating strategy is provided in the Supplementary Information.
