# Supplementary material for: Titrating bacterial growth and chemical biosynthesis for efficient N-acetylglucosamine and N-acetylneuraminic acid bioproduction
Source: Nat Commun. 2020 Oct 8;11:5078. doi: 10.1038/s41467-020-18960-1 (PMC7544899; doi:10.1038/s41467-020-18960-1)
Supplement: Supplementary file 3 — Description of Additional Supplementary Files [file 41467_2020_18960_MOESM3_ESM.pdf]

Description of Additional Supplementary Files

File Name: Supplementary Data 1

Description: Strains and plasmids used in this study.

File Name: Supplementary Data 2

Description: Primers used in this study.

File Name: Supplementary Data 3

Description: All the sequences data cited in Supplementary Data 1.
